# Mattertronics for programmable manipulation and multiplex storage of pseudo-diamagnetic holes and label-free cells

**DOI:** 10.1038/s41467-021-23251-4

**Published:** 2021-05-21

**Authors:** Sandhya Rani Goudu, Hyeonseol Kim, Xinghao Hu, Byeonghwa Lim, Kunwoo Kim, Sri Ramulu Torati, Hakan Ceylan, Devin Sheehan, Metin Sitti, CheolGi Kim

**Affiliations:** 1 Department of Emerging Materials Science, DGIST, Daegu, Republic of Korea; 2 Physical Intelligence Department, Max Planck Institute for Intelligent Systems, Stuttgart, Germany

**Keywords:** Lab-on-a-chip, Biomedical engineering

## Abstract

Manipulating and separating single label-free cells without biomarker conjugation have attracted significant interest in the field of single-cell research, but digital circuitry control and multiplexed individual storage of single label-free cells remain a challenge. Herein, by analogy with the electrical circuitry elements and electronical holes, we develop a pseudo-diamagnetophoresis (PsD) mattertronic approach in the presence of biocompatible ferrofluids for programmable manipulation and local storage of single PsD holes and label-free cells. The PsD holes conduct along linear negative micro-magnetic patterns. Further, eclipse diode patterns similar to the electrical diode can implement directional and selective switching of different PsD holes and label-free cells based on the diode geometry. Different eclipse heights and junction gaps influence the switching efficiency of PsD holes for mattertronic circuitry manipulation and separation. Moreover, single PsD holes are stored at each potential well as in an electrical storage capacitor, preventing multiple occupancies of PsD holes in the array of individual compartments due to magnetic Coulomb-like interaction. This approach may enable the development of large programmable arrays of label-free matters with high throughput, efficiency, and reliability as multiplex cell research platforms.

## Abstract (web summary)

Here, the authors present a pseudo-diamagnetophoresis mattertronic approach for programmable manipulation of label-free cells. Immersed in biocompatible ferrofluids, single cells are moved along linear negative micromagnetic patterns, switched at eclipse diode patterns and stored in potential wells.

## Introduction

In recent years, lab-on-a-chip technologies have been offering significant advantages for the manipulation and separation of particles and cells for numerous applications, including gene sequencing, disease diagnosis, and single-cell separation and analysis^1–6^. Here, single-cell separation and analysis are of critical importance in improving our understanding of intercellular heterogeneity, mechanisms of cellular functionality, as well as discovering unique characteristics of individual cells^7^. Microfluidic devices and methods work at a scale comparable to the cell size and are suitable for single-cell observation^8–10^. Previously, various types of microfluidic methods have been developed, relying on flow cytometry^11–13^, photophoresis^14–19^, acoustophoresis^20–26^, dielectrophoresis^27–30^, and magnetophoresis^5,31–33^, for the manipulation and separation of single or bulk amount of cells. Supplementary Table 1 compares such microfluidic methods in terms of labeling, precision, throughput, cellular damage, programmability, and potential applications for cells-on-chip. In comparison to these methods, magnetophoresis offers unique advantages due to the use of magnetic fields by providing remote noninvasive manipulation ability, programmability, lower heat generation, and biocompatibility and by fixing the cells during analysis and adjusting the distance between the cells. Furthermore, the magnetophoresis method provides safe and effective detection of single cells from a complex mixture for further downstream analysis and testing. In addition, magnetic field-based techniques are versatile to enable high throughput and screening of both magnetic and non-magnetic particles/cells for subsequent manipulation. Depending on the relative magnetization properties of the particles/cells in the surrounding medium, magnetophoresis can be classified as positive magnetophoresis or negative magnetophoresis^34–37^.

In “positive” magnetophoresis, the overall magnetization of the particles/cells is typically larger than that of the surrounding medium so that they move towards higher magnetic field regions^38–48^. The bulk separation of magnetic particles and labeled cells by continuous free-flow in microfluidic channels has received limited attention due to the lack of directional and individual control^49^. Therefore, the local manipulation of particles/cells has been developed by the controlled field landscape over on-chip patterned nanowires^50–53^, micromagnets^9,54–57^, and ferrite garnet magnetic films^58,59^. Magnetic thin film is a two-dimensional (2D) structure and it does not affect other additional three-dimensional (3D) structures on-chip. Hence, the on-chip microfabrication method allows easy integration of 2D and 3D structures in a single chip. Recently, the magnetophoretic circuitry concept was introduced, that is the magnetic circuits were functioning similar to the electrical conductors, diodes, and capacitors. The circuits were constructed from the combination of lithographically fabricated magnetic thin film structures and the current lines. The magnetic structures were guiding magnetizable objects to the desired locations and the current lines were used for the switching, digital control, and storage of homogeneous and heterogeneous pairing of magnetically labeled cells allowing more than one cell per compartment^5^. Thus, these compartments provide a platform for studying single cell–cell communication. However, there is a necessity to design and fabricate versatile magnetic storage compartments to store a single-cell in each individual compartment to store and study single cells over a long period. Further, integrating magnetic elements with the current lines is time-consuming and requires complex lithography fabrication steps. Thus, the design of magnetic thin film structures itself to function similarly to the electrical circuitry elements would be a great challenge. Moreover, the reported method does not allow the programmable and simultaneous manipulation of both labeled and unlabeled cells. It only allows the manipulation and separation of labeled cells from the stack of cells. Furthermore, another main drawback of this method is that the cell biomarkers must be known to enable labeling with magnetic carriers. In some cases, the discovery of biomarkers for specific cells is a critical step for making a leap in a specific research field. For example, a large leap in cancer stem cell research was possible after the discovery of CD133 as a cancer stem cell (CSC) marker. That is, the first evidence of CSCs was reported in around 1990, but advances in CSC research were limited until 2003 when CD133 was identified as a cancer stem cell marker^60^. Hence, there is a strong demand for a manipulation technology relying on a label-free method, such as “negative” magnetophoresis, to study single cells with unknown surface biomarkers.

Various studies have been conducted earlier on bulk non-magnetic particle and label-free cell manipulation^61–63^, separation^34,64,65^, focusing^66,67^, and assembly^38^ using the “negative” magnetophoresis method. For example, Zhao et al.^64^ separated label-free HeLa cells from red blood cells (RBCs) depending solely on their size. Zeng et al.^65^ sorted label-free cells from polystyrene microparticles in ferrofluids using two offset permanent magnets placed near a straight microchannel. These existing cell manipulation methods still do not satisfy precise and programmable manipulation of label-free single cells and the inability to perform multiplex cell control for local storage. Further, diamagnetic trapping and arraying of label-free Jurkat cells were achieved in a simple manner by Kauffmann et al.^68^ on square shape micromagnet arrays using different concentrations of Gd-based contrast agents. By pouring the cell suspension onto the chip, the bulk cells were trapped between the edges of the micromagnets due to the diamagnetic forces generated from the magnetic thin film layer. However, this method lacked specific cell selection and the individual transportation of cells to the trapping compartments with a digital circuit analogy. Hence, there is a strong need for a future technology enabling single-cell manipulation by a label-free method with the ability to transport the individually identified unique cells to targeted regions in a programmable circuitry manner. Precise and programmable manipulation is highly beneficial to automate (without external processing such as imaging process and programming) and place selected single cells in desired locations.

In this work, we demonstrate a digital circuitry-based manipulation and synchronous separation and storage of individual non-magnetic particles and label-free cells on newly designed negative micro-magnetic patterns equivalent to mattertronic circuitry elements. We use biocompatible ferrofluids as the medium which have much higher magnetization $({{\bf{M}}}_{{\mathrm{f}}}\left({\bf{H}}\right))$ than the magnetization of the suspended non-magnetic particles and label-free cells $({{\bf{M}}}_{{\mathrm{p}}})$ in the microfluidic environment. Therefore, these non-magnetic particles and label-free cells will exhibit an effectively large diamagnetic response in the presence of ferrofluids. Thus, by analogy with the electronic hole, herein, we call non-magnetic particles and label-free cells as “pseudo-diamagnetic (PsD) holes”. Furthermore, we devise mattertronic circuitry elements to transport, switch, and store PsD holes in magnetophoretic microfluidic platforms in a way similar to electrical conductors, diodes, and capacitors. First, by adjusting the magnetization of ferrofluids, we can achieve controllable PsD hole transportation on the conductor under moderate magnetic fields. Second, eclipse diode patterns are developed for efficient switching of different PsD holes in a reliable manner. We also test the performance of the switching diode for selection and separation of label-free THP-1 cells (human monocytic cells). Third, capacitor patterns are designed to store multiple PsD holes in one compartment, as well as single PsD holes in each compartment with multiple occupancy exclusion characteristics.

## Results

### Working principle of PsD hole manipulation

**Fig. 1:**
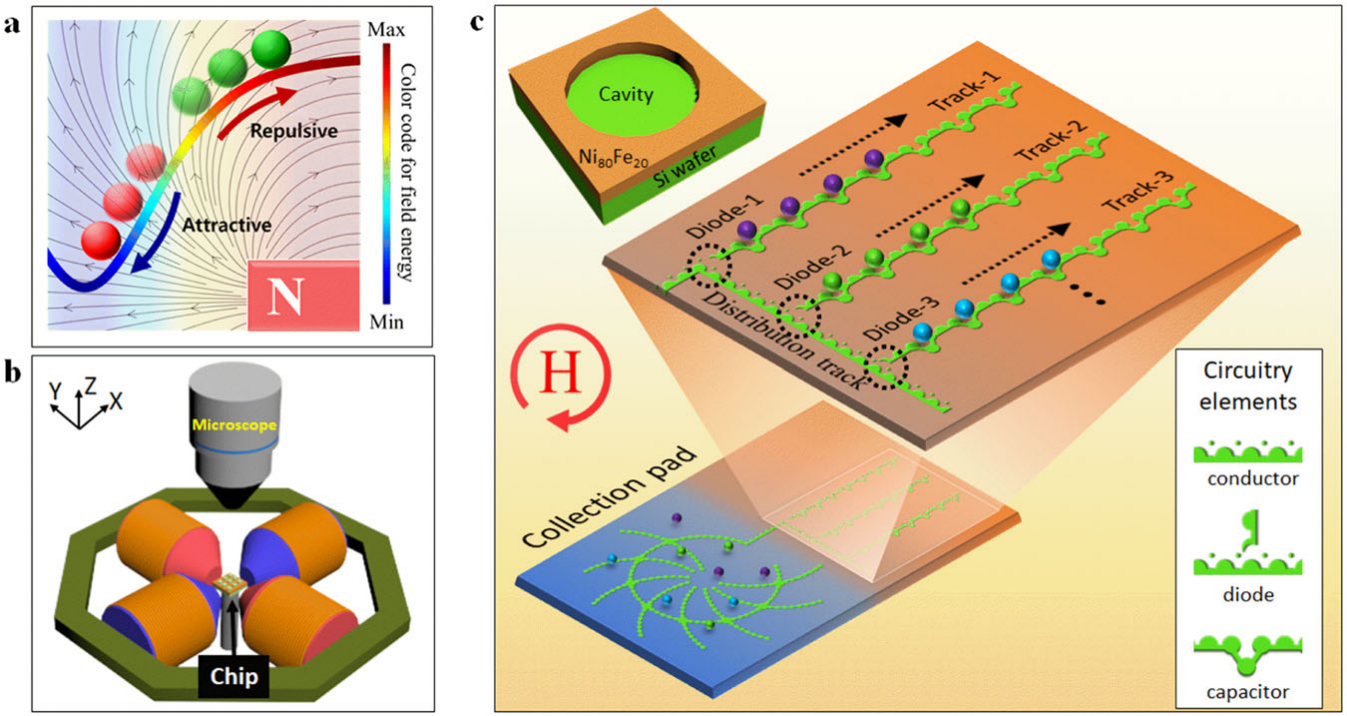
Illustration of the different sizes of PsD holes storage occupancy under magnetic fields. **a** Conceptual diagram for a PsD hole (green spheres) being pushed away from the magnet. This indicates that the PsD hole moves to a higher magnetic field energy, which is opposite to the magnetic particles (red spheres) moving towards a lower field energy. **b** Schematic diagram of the electromagnetic coil setup. The micro-magnetic chip is placed at the center, which can be observed through a microscope. **c** Schematics of the integrated platform for the collection, distribution, transportation, sorting, and storage of PsD holes. Here, thin permalloy negative micro-magnetic (mattertronic) circuitry elements perform conduction, sorting at the eclipse diode, and storage of different sizes of PsD holes in individual capacitors under clockwise rotating magnetic fields. Each track allows only one size of PsD hole through the gaps designed at the T-shaped junction. Spheres shown in purple, green, and light blue correspond to PsD holes of different sizes.

**Fig. 2:**
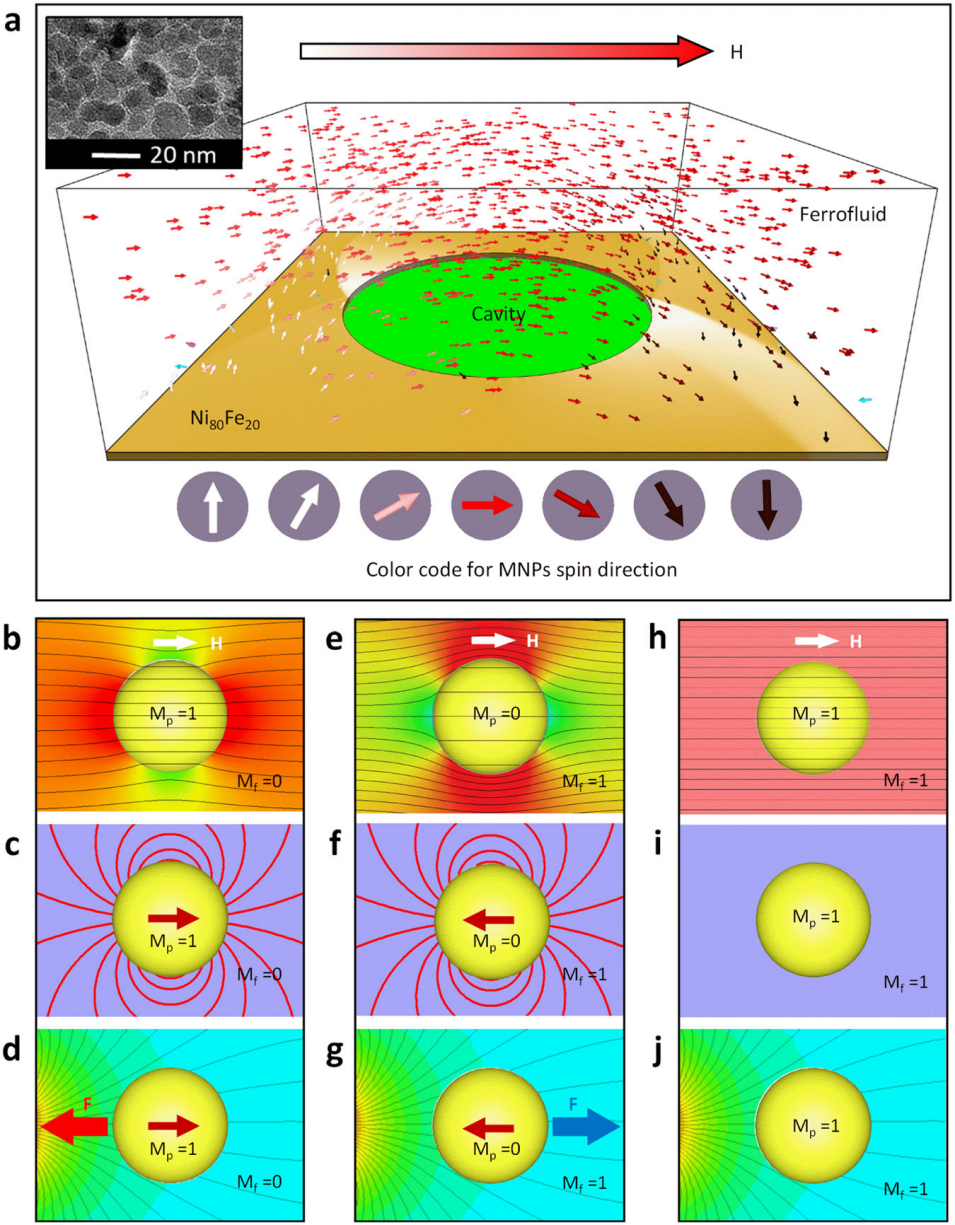
Simulation results representing particles dipole moment direction in the surrounding fluid. **a** The simulated spin directions of Fe_3_O_4_ nanoparticles over the negative micro-magnetic disk pattern. The inset presents a transmission electron microscope (TEM) image of nanoparticles with an average size of 10 nm. **b**, **e**, **h** Magnetic flux distributions for various magnetization and of a particle in a surrounding fluid corresponding to a magnetic dipole and a PsD hole, respectively. **c**, **f**, **i** Induced particle dipole moment after subtracting the applied magnetic field, revealing magnetic dipole, PsD dipole, and no dipole moment, respectively. **d**, **g**, **j** Magnetic force direction under non-uniform magnetic fields for various magnetization and of a particle in a surrounding fluid corresponding to a magnetic dipole and a PsD hole, respectively.

In general, PsD holes suspended in ferrofluids can be pushed away by an applied gradient magnetic field, as shown in Fig. 1a. In contrast, if the magnetic particles have larger magnetization than the ferrofluid, the particles will be attracted to the maximum field region. However, to obtain individual rather than bulk control of the PsD holes in the presence of ferrofluids, we have designed on-chip negative micro-magnetic patterns in the form of electric circuitry elements. The chip is placed at the center of the working space of the electromagnetic coil setup, as shown in Fig. 1b. Under a rotating magnetic field, the PsD holes conduct along with the minimum magnetic field locations on the periphery of the negative micro-magnetic patterns. The PsD holes of varied sizes can be sorted at the eclipse diodes, thereby navigating in different tracks and being stored in individual capacitors, as shown in Fig. 1c. Here, the negative micro-magnetic patterns are lithographically fabricated vacant cavities surrounded by a 100-nm-thick Ni_80_Fe_20_ magnetic layer on a Si wafer, as shown in inset Fig. 1c (see Supplementary Fig. 1 and Supplementary Note 1 for the detailed microfabrication procedures). Figure 2a shows the magnetic spin directions possessed by the ferrofluids over a negative micro-magnetic pattern under the influence of an applied field of 90 mT. A transmission electron microscope (TEM) image of the ferrofluids consisting of Fe_3_O_4_ nanoparticles is shown as an inset.

A physical model can be used to evaluate the effective dipole moment of the magnetic particles and PsD holes immersed in ferrofluids. This model employs a continuum approach to approximate the local ferrofluid magnetization, which is given by

1 $${\bf{m}}\left({\bf{H}}\right)=({{\bf{M}}}_{{\mathrm{p}}}-{{\bf{M}}}_{{\mathrm{f}}}({\bf{H}})){V}_{\mathrm{p}}$$

where ${\bf{m}}\left({\bf{H}}\right)$ is the effective dipole moment of the particle, ${V}_{\mathrm{p}}$ is the volume of the particle, and ${{\bf{M}}}_{{\mathrm{p}}}$ and ${{\bf{M}}}_{{\mathrm{f}}}{\boldsymbol{(}}{\bf{H}}{\boldsymbol{)}}$ are the magnetization of the particle and the ferrofluid, respectively.

By the application of the magnetic field along the horizontal direction, Fig. 2b clearly reveals a field distortion around a particle when ${{\bf{M}}}_{{\bf{p}}}$ and ${{\bf{M}}}_{{\mathrm{{f}}}}{\boldsymbol{(}}{\bf{H}}{\boldsymbol{)}}$ are 1 and 0, respectively. After subtracting the applied field, the net dipole moment of the particle is along the field direction (Fig. 2c) and can be quantified by Eq. (1). Also, in this case, the particle moves toward a strong magnetic region (Fig. 2d) experiencing positive magnetophoresis. When the particle and ferrofluid normalized magnetizations are assumed to be 0 and 1, respectively, the magnetic field expulsion around the particle is observed (Fig. 2e). Therefore, the particle induces a dipole moment in the opposite direction to the applied field (Fig. 2f), forming these particles as pseudo-diamagnets. In this case, the particle moves toward a weak magnetic region (Fig. 2g) experiencing negative magnetophoresis. Moreover, when both the particle and the ferrofluid normalized magnetizations are equal to 1, there will be no field distortion at all, as shown in Fig. 2h; thus, no dipole moment and the magnetic force are induced by the particle (Fig. 2i, j). Furthermore, the magnetostatic potential energy *U* of a dipole under an applied field ${\bf{H}}$ is given by

2 $${U}=-\frac{1}{2}{{\rm{\mu }}}_{0}{\bf{m}}\left({\bf{H}}\right)\bullet {\bf{H}}$$

Additionally, the magnetic driving force ${\rm{F}}$ acting on the particles inside ferrofluids experienced by the applied external magnetic field is given by

3 $${\bf{F}}={V}_{\mathrm{p}}[({{\bf{M}}}_{{\rm{p}}}-{{\bf{M}}}_{{\rm{f}}}\left({\bf{H}}\right))\bullet \nabla ]{\bf{B}}$$

where ${\bf{B}}$ is the magnetic flux density.

**Fig. 3:**
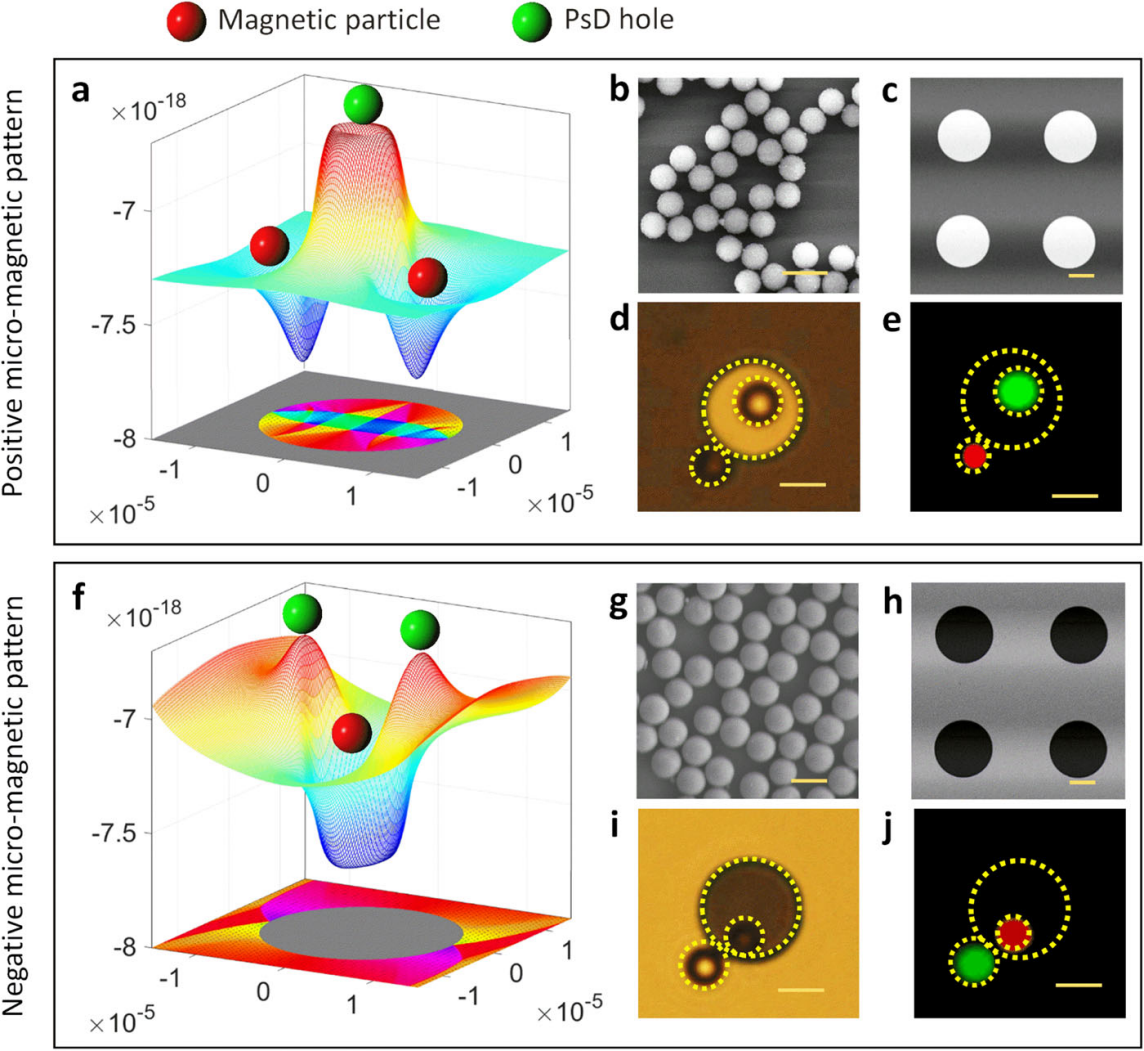
Magnetic particles and PsD holes trapping locations on positive and negative micro-magnetic patterns. **a** Magnetostatic potential energy landscape over a full-disk positive micro-magnetic pattern predicts the trapping locations of magnetic and PsD holes. The magnetic domains simulated for the positive micro-magnetic pattern under 2 mT. **b**, **c** TEM and SEM images of 2.8 µm magnetic particles and positive magnetic patterns, respectively. **d**, **e** Visualization of optical and fluorescent images of trapped magnetic particles and PsD holes, respectively, on a full-disk positive magnetic pattern. **f** Magnetostatic potential energy landscape over the full-disk negative micro-magnetic pattern predicts the trapping locations of magnetic particles and PsD holes. The magnetic domains simulated for the negative magnetic pattern under 2 mT. **g**, **h** TEM and SEM images of 3.57 µm PsD holes and negative magnetic pattern, respectively. **i**, **j** Visualization of optical and fluorescent images of trapped magnetic particles and PsD holes, respectively, on a negative micro-magnetic pattern. Scale bar: 5 µm.

Equations (1) and (2) imply that, for the magnetic particle $({{\bf{M}}}_{{\mathrm{{p}}}} > {{\bf{M}}}_{{\mathrm{f}}}({\bf{H}}))$ in ferrofluids, its potential energy is strictly negative and causes the magnetic particle to move towards the regions of the maximum field, which corresponds to the regions of the minimum magnetic potential energy (blue) shown in Fig. 3a. However, for the PsD holes $({{\bf{M}}}_{{\mathrm{{p}}}} < {{\bf{M}}}_{{\mathrm{{f}}}}({\bf{H}}))$, the potential energy is positive, so that they move towards the regions of magnetic field potential maxima (red), as shown in Fig. 3f.

Based on the magnetic phenomena similar to the behavior of electrons and holes under electric fields, we experimentally showed the locations of both the magnetic particles and PsD holes on positive and negative micro-magnetic disk patterns under an applied field of 90 mT at an operating frequency of 0.05 Hz, as shown in Fig. 3d, e, i, j. Here, the scanning electron microscope (SEM) images of the magnetic particles and the non-magnetic particles (PsD holes), as well as the positive and the negative micro-magnetic disk patterns, are shown in Fig. 3b, c, g, h. On the positive micro-magnetic pattern, the PsD holes are trapped at the disk center since the regions of magnetic field potential energy maxima (red) are positioned over the disk pattern, and the magnetic particle follows the potential energy minima (blue) located at the disk periphery, as shown in Fig. 3d, e and Supplementary Fig. 2a. In contrast, the regions of magnetic field potential energy maxima (red) are located at the periphery of the two poles on the negative pattern for trapping the PsD holes, and the potential energy minima (blue) are at the disk center, trapping the magnetic particles as shown in Fig. 3i, j and Supplementary Fig. 2b (Supplementary Movie 1).

### Ferrofluid characterization

Ferrofluids are colloidal dispersions of iron oxide magnetic nanoparticles suspended in a liquid medium^69^. The nominal diameter of nanoparticles is around 10 nm, and they are usually coated with a surfactant. Hence, when ferrofluids are exposed to a magnetic field, the magnetic attraction of nanoparticles is weak enough that the surfactant’s Van der Waals force inhibits magnetic clumping or particle agglomeration. In our experiments, the magnetization of ferrofluids is an essential parameter to manipulate the PsD holes on negative micro-magnetic patterns (see Supplementary Fig. 3 for magnetization properties of ferrofluids at different concentrations). The ferrofluid magnetization is dependent on the concentration of nanoparticles, and in turn, the magnetic force shown by Eq. (3) is a function of magnetization and size of the PsD holes. The minimum magnetic force is 0.043 pN as the PsD hole begins to move beyond the viscous force (see Supplementary Fig. 4).

**Fig. 4:**
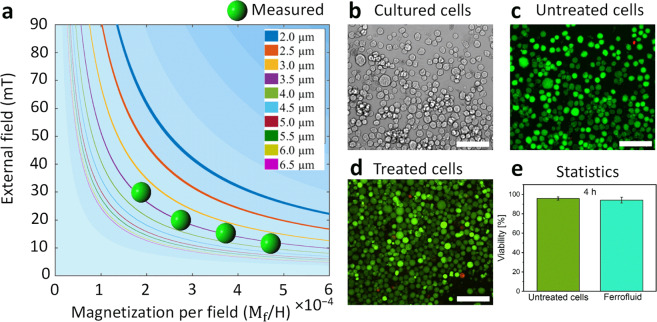
Varied magnetic field strengths depending on ferrofluid magnetization per field and the analysis of cell viability with and without the presence of ferrofluid. **a** Plot of magnetic field strength and different ferrofluid magnetization per field (${{\bf{M}}}_{{\rm{f}}}/{\bf{H}}$) for PsD hole movement for various particle sizes. The line interval corresponds to the 0.5 µm radius of the PsD hole. Smaller size PsD holes require higher magnetic fields and magnetizations. **b**, **c** Differential interference contrast (DIC), live (green) and dead (red) fluorescence images of THP-1 cells in the cell culture medium without ferrofluid treatment. **d** Live and dead fluorescence images of THP-1 cells after 4 h of treatment with ferrofluid. Scale bar: 100 µm. **e** Quantitative analysis of the viability of THP-1 cells demonstrating no acute toxicity induced by ferrofluid. Data are presented as the mean ± standard deviation (SD).

Furthermore, we experimentally determined the critical magnetic field strength required to manipulate the 3.57 µm PsD holes on a negative micro-magnetic disk pattern at different ferrofluid magnetizations per field ${{\bf{M}}}_{{\mathrm{f}}}{\boldsymbol{/}}{\bf{H}}$, as shown in Fig. 4a. Here, the critical magnetic field denotes the field at which the PsD holes start to acquire the strength to move in the phase-locked motion^14^ with respect to the field direction. Below the critical field, the PsD holes cannot follow the field direction. The PsD holes require higher magnetic field strengths to move on the periphery of the pattern as the ferrofluid concentration decreases, which is denoted by contours of the equi-force lines (shown by solid lines) calculated by using Eq. (3). There is a good agreement between measured values from different ${{\bf{M}}}_{{\mathrm{f}}}$**/H** values and the calculated critical fields. From the experimental evidence, the 3.57 µm PsD holes are in the phase-locked motion at 30 and 10 mT for ${{\rm{M}}}_{{\rm{f}}}$/H of 2 × 10^−4^ and 4.8 × 10^−4^, respectively.

### Cell viability

The viability of the non-adherent THP-1 cells inside the diluted ferrofluid (0.2% v/v) was investigated over the course of 4 h. See Supplementary Fig. 5 for the cell viability of adherent HUVECs. There was no significant difference found between these two types of cells in terms of viability. Differential interference contrast (DIC) optical microscopy and fluorescence microscopy images confirmed the viability and morphology of the THP-1 cells treated with ferrofluids to be comparable to those of native cells in the cell culture plate (Fig. 4b–d). For fluorescence imaging, we treated the cells with calcein acetoxymethyl ester (calcein-AM) and propidium iodide fluorescent probes. Inside live and healthy cells, intracellular esterase activity converts calcein-AM (cell penetrable) to calcein, which is retained inside the cells and produces intense fluorescence upon binding intracellular Ca^2+^. However, when the cells start losing their intact cellular membrane, propidium iodide (cell impenetrable) is able to diffuse into the cells from the pores and chelate to the genomic DNA to fluoresce.

Both THP-1 cells treated with ferrofluids and the untreated culture group exhibited similar patterns of green and red fluorescing cells, corresponding to live and dead cells, respectively (Fig. 4c, d). We determined that there was no significant difference between the viability of the treated and untreated cells, as shown in Fig. 3e. Thus, cellular viability tests reveal a safe window for potential separation and trapping analysis applications based on the size and magnetization of the cells. Currently, we have limited the viability of cells and the usage of the entire system to 4 h (see Supplementary Fig. 6 for cell viability up to 24 h with different ferrofluid concentrations). If needed for prolonged hours, a major future challenge would be to adjust the basic necessary culture conditions, including the nutrients, oxygen support, and carbon dioxide removal from a small analysis volume, to sustain the cellular viability. In addition to the cellular viability, we tried to understand the potential impact of the ferrofluid formulation on the characteristic marker expression and proliferation of THP-1 cells. See Supplementary Figs. 7 and  8 and Supplementary Notes 2 and  3 for the detailed experimental results.

### Circuitry elements for PsD hole transportation

**Fig. 5:**
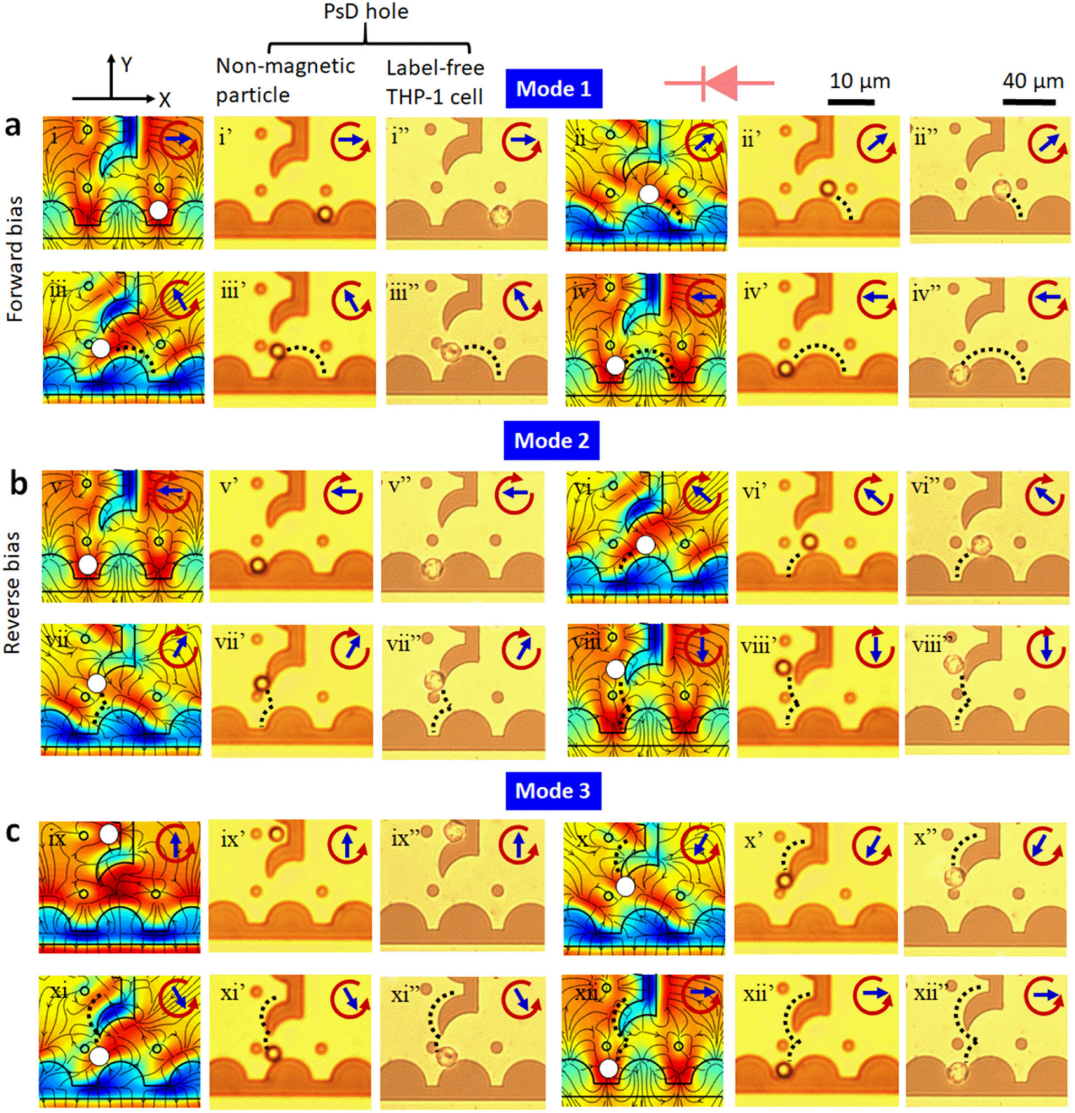
Experimental and simulation results showing the traces of PsD holes and cells during magnetic field rotations. Magnetic force lines and the field potential energy maxima (red) landscapes (first column) correspond to traces of 3.57 µm PsD holes in diameter (second column) and THP-1 cells (third column) over a negative micro-magnetic eclipse diode under three-moving modes. **a** Mode 1 in forward bias mode. Due to the merging of maximum potential energies, the PsD hole located at the right side of the eclipse junction switches the junction gap *d* = 7 µm during counterclockwise field rotation at angles *ɵ*  = 180°, 240°, 300°, and 360°. Blue arrows represent the direction of the magnetic field. The black dotted lines denote the PsD hole/label-free cell trajectories on the eclipse diode. **b** Mode 2 in reverse bias mode. The PsD holes/label-free cells located on the left side of the eclipse junction cannot switch the eclipse junction, thereby navigating towards the *Y*-axis due to the potential energy split under clockwise field rotation at angles of *ɵ* = 360°, 300°, 240°, and 180°. **c** Mode 3, the PsD holes/label-free cells jump from the positive *Y*-axis to the negative *X*-axis at an eclipse junction during counterclockwise field rotation at angles of *ɵ* = 90°, 240°, 300°, and 360°.

The manipulation of PsD holes suspended in ferrofluids is determined primarily by the magnetic force balance and magnetostatic potential energy configurations on the mattertronic circuitry elements. Under the rotating magnetic field, PsD holes can be navigated along the linear negative micro-magnetic half-disk pathway referred to as conductor (Supplementary Fig. 9 and Supplementary Movie 2). Furthermore, similar to an electrical diode, we design the eclipse diode pattern with the arrangement of the conductor pathways along horizontal and vertical tracks in the *X*-axis and *Y*-axis directions to switch and separate PsD holes. Figure 5 presents the magnetic force and the potential energy landscape to govern the movement direction of the PsD holes over the eclipse diode pattern, where the directions of the magnetic fields are indicated by blue arrows.

When the PsD holes move towards the negative *X*-axis under the counterclockwise rotating field, they cross the eclipse junction due to the merging of maximum potential energies, leading to continuous motion towards the negative *X*-axis, as shown in mode 1 (Fig. 5a). This behavior is the same as the conventional current flow in the forward bias mode of an electrical diode. Here, the first column corresponds to different field directions and potential energy configurations, and the second and third columns are optical images of the moving non-magnetic particles and THP-1 cells (PsD holes), respectively. Moreover, in mode 2, when the PsD holes move along the positive *X*-axis direction and reach the eclipse junction during the clockwise rotating field, they cannot switch to the right side of the junction. However, since the maximum magnetic potential energy splits into two energy peaks, the PsD holes switch towards the higher energy peak at the *Y*-axis, as shown in Fig. 5b. It corresponds to no current flow in the reverse bias mode of an electrical diode. Furthermore, the PsD holes located at the *Y*-axis jump from the eclipse junction to *X*-axis and move towards the negative *X*-direction during the counterclockwise rotating field in mode 3, as shown in Fig. 5c (Supplementary Movie 3).

### Switching efficiency of PsD holes at eclipse diode with anti-dot

**Fig. 6:**
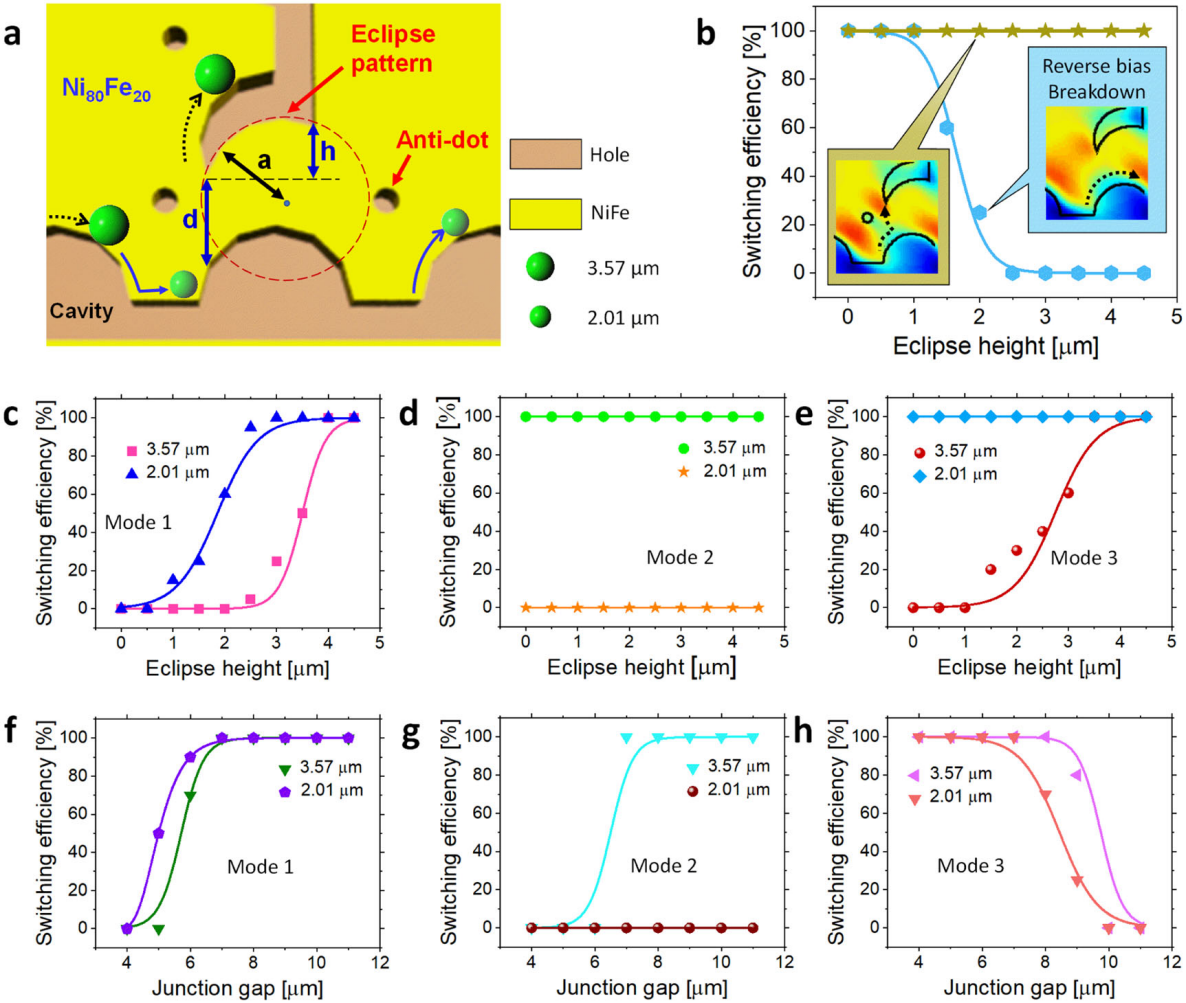
Switching efficiency of PsD holes on eclipse diode patterns. **a** Schematic diagram representing the geometrical construction of the eclipse diode with an anti-dot 2 µm in diameter. Here, “*a*” is the radius (5 µm) of the disk pattern, “*d*” is the junction gap (7 µm), and “*h*” is the eclipse height (4 µm). **b** Switching efficiency comparison for 3.57 µm PsD holes with and without an anti-dot. The diode integrated with an anti-dot acquires 100% switching efficiency at all eclipse heights, whereas the absence of an ant-dot leads to zero switching efficiency above 2 µm eclipse height. **c**–**e** Switching efficiency of 2.01 and 3.57 µm PsD holes at various eclipse heights (*h*) on an eclipse diode with an anti-dot pattern. **f−h** Switching efficiency of 2.01 and 3.57 µm PsD holes at various junction gaps (*d*) on an eclipse diode with an anti-dot pattern.

The schematic design of the eclipse diode integrated with an anti-dot is shown in Fig. 6a. The eclipse curvature at the junction is crucial for the switching of PsD holes along the negative micro-magnetic patterns. In addition, the geometrical parameters play prominent roles in separating PsD holes with different sizes. Here, “*a*” is the radius of the disk pattern, “*d*” is the gap at the T-shaped junction, and “h” is the eclipse height. It is noteworthy that the additional anti-dot is designed to enhance the switching of PsD holes from the horizontal track to the vertical track during the clockwise rotating field. Due to the distribution of potential energy at the eclipse junction, 3.57-µm-diameter PsD holes switch towards the *Y*-axis and acquire 100% switching in the presence of an anti-dot. However, in the absence of an anti-dot, switching occurs only at an eclipse height below 1 µm, as shown in Fig. 6b, which corresponds to the incomplete performance of the diode in its reverse bias mode.

The switching efficiency of PsD holes on the eclipse diode with an anti-dot is measured at various eclipse heights (*h*) from 0 to 4 µm by fixing the junction gap (*d*) at 7 µm, as shown in Fig. 6c–e. Under mode 1, 2.01 µm PsD holes have zero switching efficiency at eclipse heights of 0 and 0.5 µm, and gradual increases are observed up to 2.5 µm; thereafter, the PsD holes achieve 100% switching efficiency. For the 3.57 µm PsD holes, the switching efficiency is zero up to 2 µm eclipse height and 100% from 4 µm, as shown in Fig. 6c. Under mode 2, 2.01 µm PsD holes have zero switching efficiency, whereas the 3.57 µm PsD holes have 100% switching efficiency at all eclipse heights, as shown in Fig. 6d.

Under mode 3, there is 100% switching efficiency at all eclipse heights for 2.01 µm holes, and the 3.57 µm PsD holes have zero switching efficiency from eclipse heights of 0–1.5 µm. However, as the eclipse height increases, the switching efficiency gradually increases to a maximum at an eclipse height of 4.5 µm, as shown in Fig. 6e. Indeed, the switching efficiency at different gaps is observed by keeping the eclipse height constant at 4 µm, as shown in Fig. 6f–h. The abovementioned results show that the junction gaps and eclipse heights, 7 and 4 µm, respectively, provide the best suitable combination to obtain 100% local switching efficiency similar to the forward and reverse bias modes of an electric diode.

### High-throughput storage capacitors of PsD holes

Magnetophoretic cell manipulation and storage is divided into two methods: continuous and parallel processing.

**Fig. 7:**
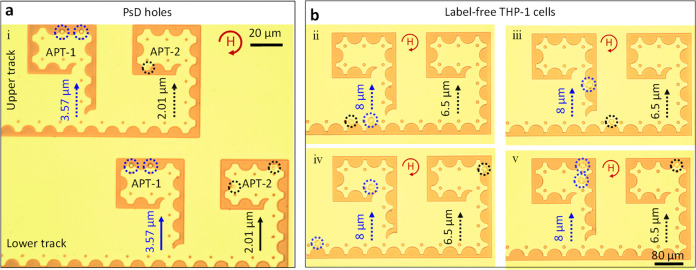
Capacitors to store multiple PsD holes and cells. **a** Capacity storage of multiple PsD holes in single compartments arranged along upper and lower tracks. PsD holes 3.57 µm (blue circles) and 2.01 µm (black circles) in diameter occupied APT-1 and APT-2, respectively. **b** Bright-field images of THP-1 cells 8 µm (blue circles) and 6.5 µm (black circles) in diameter sorted at the eclipse junctions and delivered to storage apartments APT-1 and APT-2, respectively. The curved arrow in red denotes the clockwise rotating magnetic field.

*Continuous processing*: This method provides control of the cell motion over relatively long distances of travel under a constant rotating magnetic field. In this study, the cells can be effectively separated at the eclipse junction based on their size and immediately sent to different tracks for local storage enabling continuous processing. Size-dependent cell sorting can be equipped with the identification of monocytes, particularly monocyte to macrophage maturation. In order to define a differentiated monocyte as a monocyte-derived macrophage, hallmark functional features of macrophages should be assessed, such as enhanced size, lysosomal protease expression, and phagocytic capabilities. Separation of cells based on their size can enable purification and further analyses of the individual groups. Thus, the square-shaped compartments similar to an electrical capacitor are designed with the integration of negative micro-magnetic eclipse diode patterns (Fig. 7 and Supplementary Fig. 10) for the size-dependent sorting and storage of label-free cells of 8 and 6.5 µm in diameter. An array of compartments APT-1 and APT-2 are arranged in the upper track and lower tracks in consideration of the abovementioned switching efficiency modes. The sorting of PsD holes occurs at an eclipse junction with *d* = 7 µm (Mode 2, Fig. 5b), in which 3.57 µm PsD holes switch towards the *Y*-axis and 2.01 µm PsD holes move along the *X*-axis. Furthermore, 3.57 and 2.01 µm PsD holes enter (Mode 1, Fig. 5a) APT-1 and APT-2, respectively, as shown in Fig. 7a. The PsD holes will only move inside the compartment (Mode 3, Fig. 5c), leading to permanent storage. Moreover, label-free THP-1 cells can also be sorted and stored in compartments based on their size and geometrical parameters of the negative micro-magnetic pathways, as shown in Fig. 7b (Supplementary Fig. 11 and Supplementary Movie 4). In addition to the square-shaped compartments, we have shown additional designs of storage compartments in Supplementary Fig. 12. However, this type of compartments allows storage of multiple label-free cells which could serve as a platform for the investigation of cell-pair interactions.

In addition, for single-cell research, we have developed a high-efficiency large array of storage compartments, (capacitors) in which each compartment is dedicated to a single PsD hole, i.e., a capacitor with multiple occupancy exclusion characteristics. The key mechanism for this storage is the magnetic coulomb-like interaction between two PsD holes. When the second PsD hole comes in contact with the first hole, which is in the state of occupancy, the second hole cannot enter the storage compartment, revealing multiple occupancy exclusion characteristics.

**Fig. 8:**
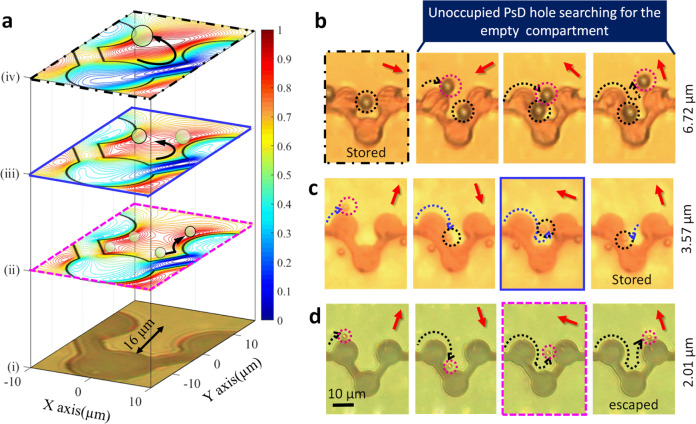
Individual storage capacitors of PsD holes. **a** Simulated potential energy distributions considering the depth of the U-shaped storage apartment (i) and the diameter of PsD holes: (ii) 2.01 µm, (iii) 3.57 µm (iv), and 6.72 µm. The potential energy planes plotted at different heights from the pattern surface. **b** Individual storage apartments having a single maximum potential energy occupied with a 6.72 µm PsD hole. Due to the magnetic coulomb-like interaction between two holes, an unoccupied PsD hole recognizes the occupied apartment and further navigates to the search for an empty apartment. **c** A 3.57 µm PsD hole permanently stored and moving back and forth between two maximum potential energy peaks inside the individual compartment. **d** PsD holes 2.01 µm in diameter that escaped from the storage compartment due to the movement of potential energy along the pattern boundary under rotating fields.

However, the occupancy of PsD holes in the compartments depends on the size of PsD holes with the potential energy distribution. Thus, we analyze the distribution of potential energies based on three different sizes of PsD holes, 2.01, 3.57, and 6.72 µm, on the capacitor micro-magnetic pattern (Fig. 8a). From the simulations, it is obvious that the 2.01 µm PsD holes have four maximum potential energy peaks (red-contour), whereas 3.57 and 6.72 µm have two-peak and single-peak maximum potential energy distributions, respectively, inside the storage compartments. Hence, 2.01 µm PsD holes sequentially follow the maximum energy peaks along the pattern boundary to escape from the storage compartment, as shown in Fig. 8b. Furthermore, 6.72 and 3.57 µm PsD holes can be stored permanently inside the U-shaped compartment with the deep potential well due to the lack of switching from one maximum energy point to the other, as shown in Fig. 8c, d, respectively. Here, the dotted black and pink circles denote the occupied and unoccupied PsD holes, respectively.

**Fig. 9:**
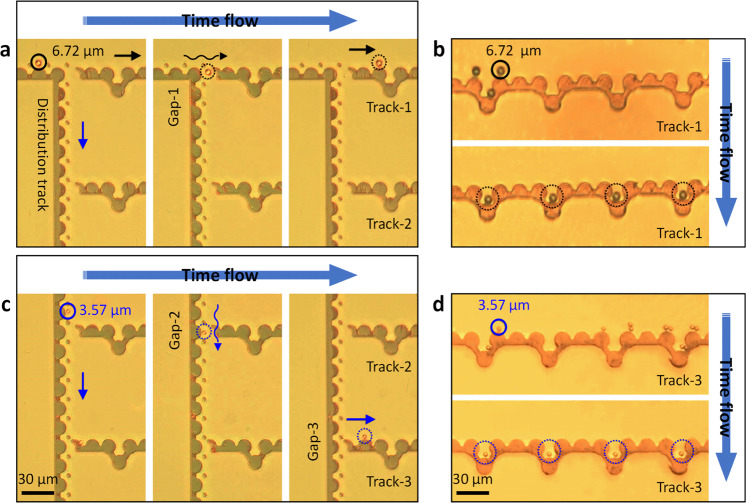
Size-dependent sorting and storage of PsD holes. Sorting of PsD holes in reverse bias mode across an eclipse diode along the distribution tracks and local storage of single PsD holes in an array of compartments along storage tracks. **a**, **b** Separation of 3.57 and 6.72 µm PsD holes by eclipse diode, where 3.57 µm PsD holes crosses Gap-1 (*d* = 15.6 µm) and then navigate towards Gap-2 (*d* = 14.3 µm), but 6.72 µm PsD holes enter Track-1 due to the lack of current flow in reverse bias mode. **c**, **d** Snapshots of transportation and occupancy of single 6.72 and 3. 57 µm PsD holes in all the individual storage compartments along Track-1 and Track-3, respectively.

An integrated platform composed of a conductor, diode, and capacitor for the synchronous sorting and storage of single PsD holes in each individual compartments is shown in Fig. 9. The storage compartments are arranged in multiple tracks. Depending on the size of the PsD holes, they move to the corresponding tracks, which leads to local separation. In addition, the PsD holes entry to each track is controlled by various junction gaps (see Supplementary Fig. 13 for junction gap parameters at each track). Because of the immediate separation of PsD holes at the junction gaps, the distance required for separation can be dramatically reduced compared to other existing methods, such as droplet-based^70^ and cell-separation methods using microfluidic channels^37^. These methods require on the order of a few millimeters of separation distance, whereas our technique just requires a separation distance on the order of a few micrometers. This unique advantage affects the structure density in a lab-on-a-chip device. Further, under rotating magnetic fields of 90 mT with 0.01 Hz, PsD holes 6.72 µm in diameter follow mode 2 (no current flow in reverse bias mode) at the eclipse diode. Hence, they are sorted into Track-1 (denoted with black arrows), as shown in Fig. 9a. The PsD holes permanently occupy empty compartments having U-shaped deep potential wells in Track-1, and the other PsD holes search for the next empty compartment until it is found, as shown in Fig. 9b. In addition, the PsD holes enter different tracks in the absence of anti-dots (see Supplementary Fig. 14). Next, the diode with Gap-3 prevents the current flow, thereby switching 3.57 µm PsD holes to Track-3 to serially occupy the storage compartments, as shown in Fig. 9c, d. Due to the potential energy distributions of the PsD holes, they just oscillate inside the compartment rather than leaving the compartment (see Supplementary Movie 5). The reliability of individual PsD hole occupancy is verified with 25 different PsD holes, resulting in 96% accuracy. Furthermore, the throughput of our individual cell storage device is limited by the concentration of the input sample and the size of the device, and the manipulation area on-chip. Thus, if the device is further optimized and if the microstructures are fabricated on a standard 100-mm silicon wafer, we estimate these arrays could be parallelized to handle more than 1 × 10^6^ cells per hour.

**Fig. 10:**
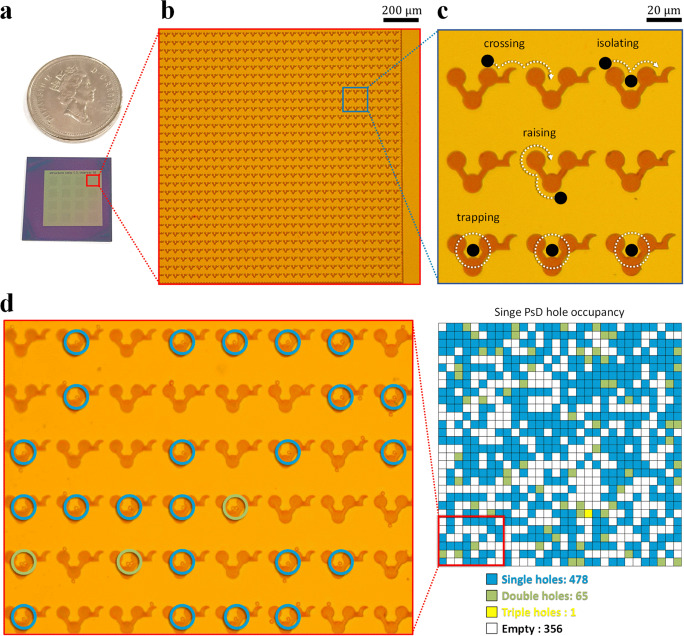
Throughput efficiency of PsD holes. **a** The microfabricated chip contains 14,400 storage compartments in a 4 × 4 array. **b** Optical image of the micro-magnetic arrays consists of 900 storage compartments. **c** Magnified optical image of the U-shaped storage compartments. **d** Optical image showing the storage of PsD holes in multiple compartments.

*Parallel processing*: In parallel processing, the cell manipulation tracks on-chip split into different parts and all the cells behave the same in the area where the constant magnetic fields are applied (see Supplementary Movie 6). We designed a 4 × 4 array of magnetic patterns (Fig. 10a) capable of storing 14,000 cells per run. However, due to the limitations of the optical microscope, we present a series of parallel magnetic tracks consists of 900 individual storage compartments. The design features 30 parallel magnetic tracks and they are separated by the optimized distance of 10 µm as shown in Fig. 10b. Moreover, each individual storage compartment is designed after the junction gap *d* = 10 µm at the eclipse junction. This eclipse junction allows the passage of PsD holes from the lower track to the upper track resulting in the efficient distribution of single cells without clogging as shown in Fig. 10c–e. By counting PsD holes from the optical images, it is determined that 478 single PsD holes, 65 double PsD holes, and 1 triple PsD hole were stored in each individual storage compartment as shown in Fig. 10f. Although we focused here on a particular area of the chip, dozens of identical microstructures can also be designed in a single chip to significantly improve high throughput.

## Discussion

We have demonstrated a mattertronic approach for the digital circuitry manipulation of single label-free matters on negative micro-magnetic patterns under moderate magnetic fields. In a similar way to electrical conductors, diodes, and capacitors, we can directionally transport, selectively switch, and locally store label-free matters in a programmable manner. In particular, we have shown the concept of storing label-free cell pairs, which could be helpful for the analysis of cell–cell communications over long times. Intercellular communication is vital for the development and regulation of functional tissues and cell growth. Further, we have developed an efficient magnetophoretic device for selective storage of individual label-free matters towards the purpose of providing single-cell level information. Thus, this novel platform could introduce a new era in the field of digital magnetophoresis for the manipulation of label-free single cells, which can overcome the drawback of current magnetophoresis requiring specific biomarkers for the target cells.

## Methods

### Materials

EMG 700 series water-based ferrofluids were purchased from Ferrotec Co., Bedford, NH, USA. The commercially obtained non-magnetic beads, which are amino-functionalized polystyrene particles (SPHERO^TM^) of sizes 2.01 μm (polymer beads AP-20-10), 3.57 μm (polymer beads AP-35-10), and 6.72 μm (polymer beads AP-60-10) and streptavidin-coated superparamagnetic particles 2.8 μm in diameter (Dynabeads M-280), were purchased from Invitrogen, Grand Island, NY, USA. The magnetic and non-magnetic particles were coated with Atto-520 Biotin and Alexa Fluor® 488 carboxylic acids so that their movements could be tracked by fluorescence microscopy. Silicon wafers coated with 200 nm SiO_2_ were purchased from Wafermart. Photoresist (AZ 5214-E) and developer were obtained from AZ Electronic Materials.

### Experimental setup

The permalloy micro-magnetic patterns required for the digital circuitry control were prepared on the bulk Si wafer using photolithography and sputtering deposition techniques. PsD hole/label-free cell manipulation was achieved by applying an external rotational magnetic field generated from an electromagnet consisting of four solenoid coils that produce uniform magnetic fields up to 250 mT. The input magnetic fields and frequencies were controlled by LabView software.

### Data analysis

The magnetic hysteresis measurements of ferrofluids were performed using a Lake-shore 7400 series vibrating sample magnetometer (VSM). Scanning electron microscopy Images of micro-magnetic patterns were obtained using Hitachi Co., model S‐4800. Transmission electron microscopy Images of ferrofluids were achieved by Hitachi Co., model HF-3300. Particle motions were tracked by video microscopy using an IMC-1040FT video camera.

### Cell culture and viability test

The THP-1 cells were obtained from the ATCC and used without further characterization. The cells were cultured in DMEM supplemented with 10% FBS, 2 mM l-glutamine, and 1% penicillin/streptomycin. Cells were incubated under standard tissue culture conditions, i.e., 37 °C and 5% CO_2_. For the viability experiment, THP-1 suspension cells were split into 96-well plates at a density of 1 × 10^4^ cells/well mixed with the magnetic fluid solution in 1× PBS. At each time point, WST-8 solution was added to each well as instructed by the supplier. The mixture was incubated at 37 °C for another 2 h followed by absorbance measurement at 460 nm. For microscopy imaging, an independent experiment with the same experimental cells was treated with 1 mM calcein-AM. The cells were imaged under a Nikon Instrument, Inc., Eclipse Ti-E.

### Flow cytometric characterization of THP-1 cells

Flow cytometric analysis were performed using the FACS BD Fortessa XR-20 (Becton Dickinson, Oxford, UK). THP-1 samples were incubated in the dark with APC Mouse Anti-Human CD16 Clone B73.1 (5 µl/Mio cells; BD Pharmingen™) and FITC Mouse Anti-Human CD14 Clone M5E2 (20 µl/Mio cells; BD Pharmingen™). After gentle washing, cells were analyzed by flow cytometry. In all measurements threshold voltage was set using unstained controls. For correct compensation antigen-specific antibodies were used. To ascertain that the cells were stained positive for the particular surface marker (e.g. CD16) in test samples, all the two surface markers were combined and the samples were acquired using the same voltage settings and compensation parameters as done for a combination of their respective controls. In total, 5000 events were analyzed by measurement and separated according to their CD16/CD14 expression profile on the cell surface in post acquisition analysis. THP-1 cells were gated based on forward scatter and side scatter characteristics. In addition, THP-1 cells were defined according to their CD14 and CD16 expression profile.

### Micro-magnetic simulations

Initially, spin configurations were performed based on pattern geometry using GPU accelerated Mumax^3^ software. We assigned some parameters such as saturation magnetization Ms = 830 emu/cm^3^ and exchange stiffness constant *A* = 13 × 10^−12^ J/m for a 100-nm-thick permalloy film under applied fields of 90 mT for effective calculations. Furthermore, by importing spin configurations into MATLAB, we achieved magnetic potential energy and force calculations.

### Reporting summary

Further information on research design is available in the Nature Research Reporting Summary linked to this article.

## Supplementary information

Supplementary Information

Description of Additional Supplementary Files

Supplementary Movie 1

Supplementary Movie 2

Supplementary Movie 4

Supplementary Movie 3

Supplementary Movie 5

Supplementary Movie 6

Reporting Summary

## Data Availability

All data generated or analyzed during this study are included in the published article and its Supplementary Information and are available from the corresponding author on reasonable request.

## Code availability

The Mumax^3^ and MATLAB codes used in this study are available from the corresponding author on reasonable request.
