# Supplementary material for: Mattertronics for programmable manipulation and multiplex storage of pseudo-diamagnetic holes and label-free cells
Source: Nat Commun. 2021 May 21;12:3024. doi: 10.1038/s41467-021-23251-4 (PMC8139950; doi:10.1038/s41467-021-23251-4)
Supplement: Supplementary file 1 — Supplementary Information [file 41467_2021_23251_MOESM1_ESM.pdf]

Supplementary Information

Mattertronics for programmable manipulation and multiplex storage of pseudo-diamagnetic holes and label-free cells

Sandhya Rani Goudu[1,2,#], Hyeonseol Kim[1,#], Xinghao Hu[2], Byeonghwa Lim[1], Kunwoo Kim[1], Sri Ramulu Torati[1], Hakan Ceylan[2], Devin Sheehan[2], Metin Sitti[2], CheolGi Kim[1]

[1] Department of Emerging Materials Science, DGIST, Daegu 42988, Republic of Korea

[2] Physical Intelligence Department, Max Planck Institute for Intelligent Systems, 70569 Stuttgart, Germany

[#] These authors contributed equally to this work.

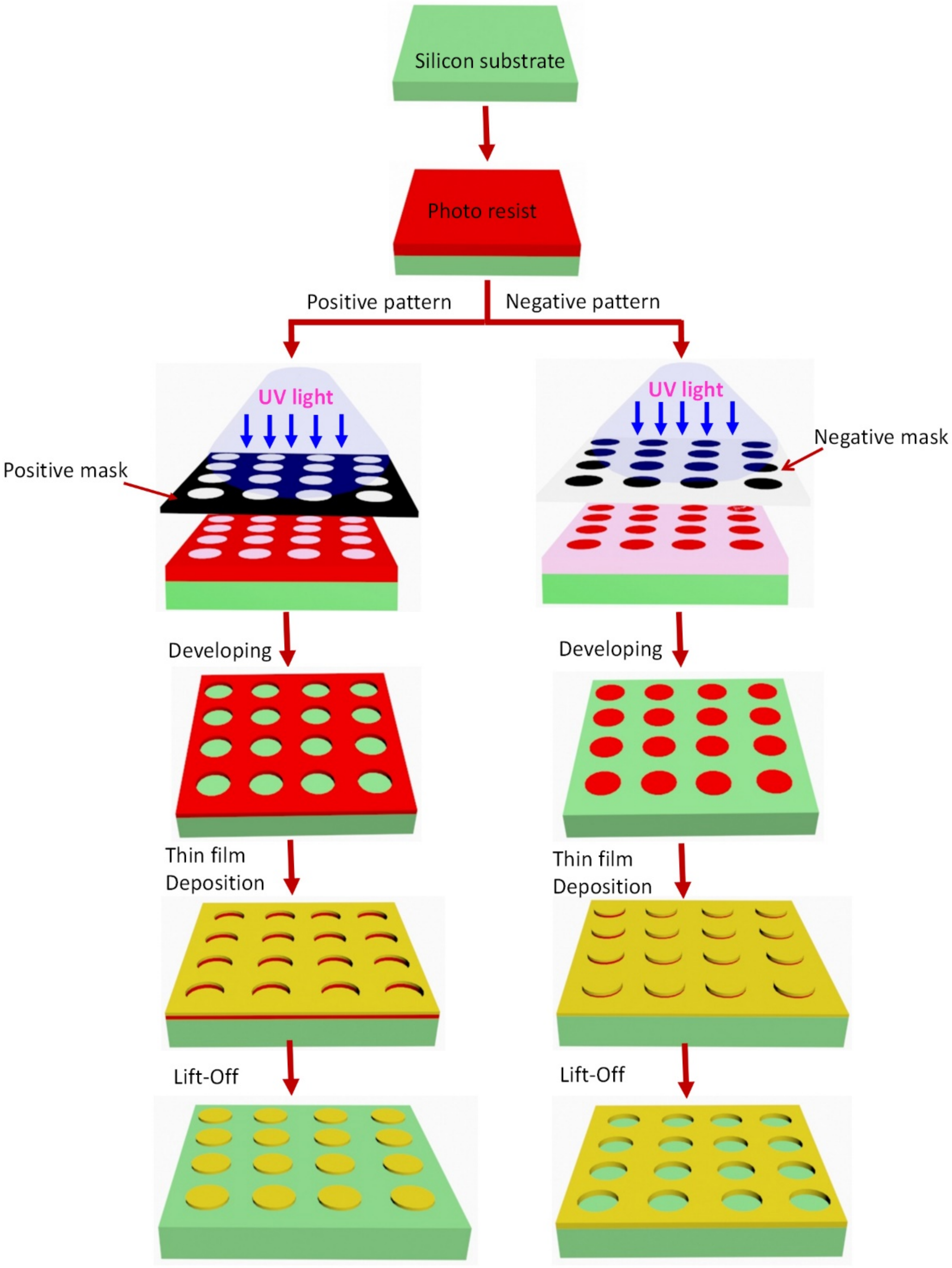

Supplementary Figure 1. Schematic representation of lithography fabrication steps of positive and negative micro-magnetic patterns.

Supplementary Note 1: Lithography fabrication process.

Photolithography: Supplementary Figure 1 shows the schematic representation of the fabrication process of the micro-magnetic patterns which can be used as the magnetic pathways to the particles. The designs were first drawn in AUTOCAD software and then they are transferred to chrome mask using electron beam lithography. The designs further can be processed onto Si wafer using photolithography process.

The Si wafers were first cleaned to remove typical contaminants, dust and bacteria. The dimensions of a typical dust particle can be anything between 1 to 50 micrometer. Thus, it is necessary to soak the wafers in acetone and methanol for 60 min using ultrasonic agitation. Further, AZ-5214 E photoresist which is a special class of photo sensitive polymer coated on the clean wafers using spin coater. The spin coater can be used to coat uniform thickness normally at 3000 – 6000 rpm for 15 – 30 seconds. By spin coating, it is possible to create thin layers even on hydrophobic surfaces. The photoresist layers can be used to obtain precise pattern formation and to protect the substrate from chemical attack during the fabrication process. The photoresist coatings can be image-able only after soft baking because the remnant solvents to be removed before exposure. Thus, the substrate is heated up on hot plate for several seconds. When the photoresist is exposed to UV light, the structural degradation occurs and the longer polymer chains break to smaller chains and washed away by a developer solution. The developer dissolves the polymer from the areas depends on the polymer type. An exact copy of the photomask will be created on the resist layer.

Lift-off method:

The thin film layer is deposited onto the substrate with lithographic photoresist pattern, covering the patterned photoresist and areas in which the photoresist has been cleared. During the lift-off

process, the sputtered sample is soaked in acetone for a while so that the remaining photoresist will be removed along with the thin film. This gives the sample only with the magnetic layer which forms the magnetic tracks on the substrate.

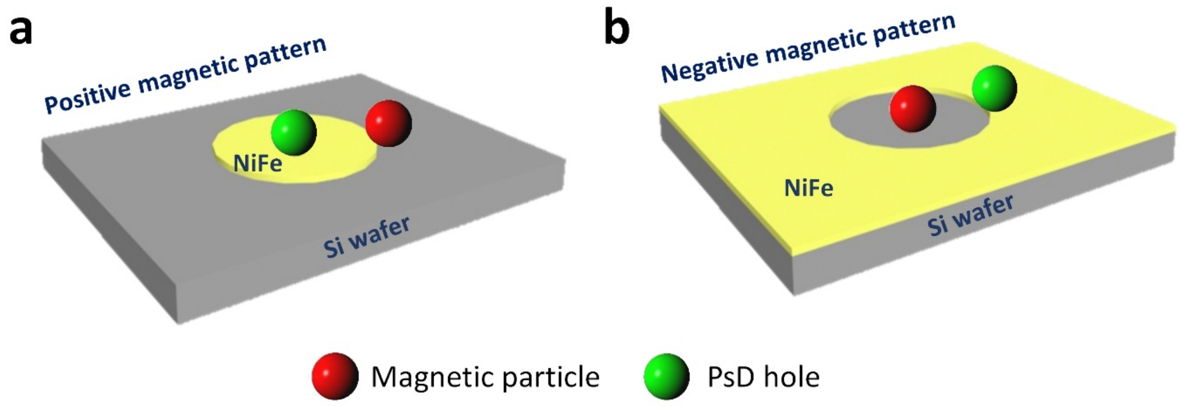

Supplementary Figure 2. Schematic representation of the locations of magnetic particles and PsD holes on positive and negative micro-magnetic disk patterns. **a** Positive micro-magnetic patterns are the lithographically fabricated magnetic structures consists of $Ni_{80}Fe_{20}$ layer, in which the PsD holes are trapped at the disk centre and the magnetic particles are located at the disk periphery. **b** Negative micro-magnetic patterns are the patterned vacant cavities surrounded by $Ni_{80}Fe_{20}$ magnetic layer on a Si wafer. The PsD holes are located at the periphery of the disk pattern. The magnetic particles are positioned above the disk pattern.

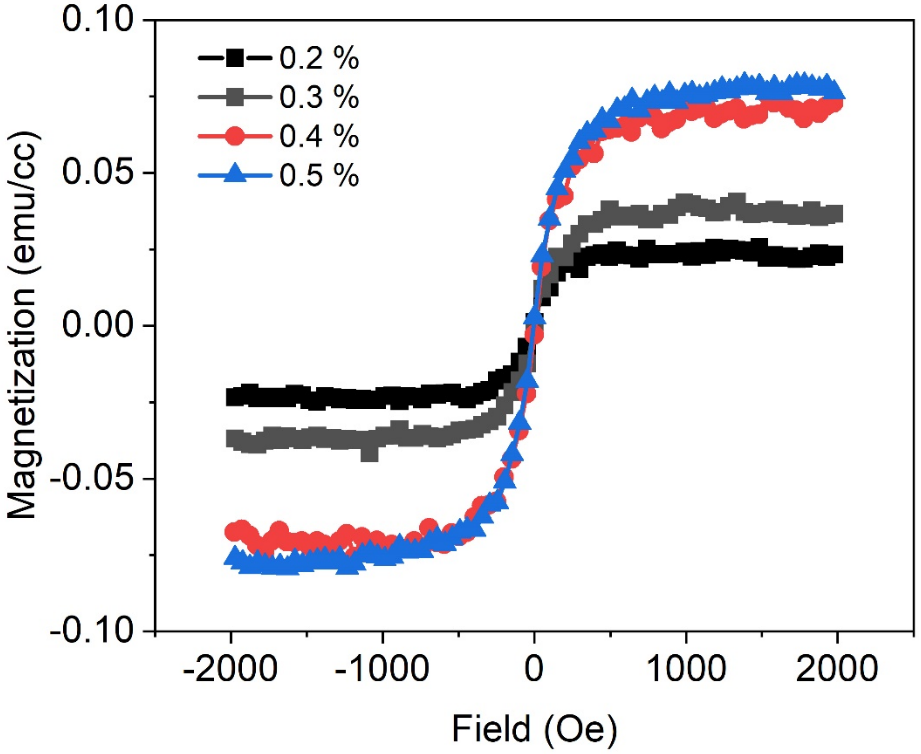

Supplementary Figure 3. Magnetic hysteresis measurement of ferrofluids at different concentrations using a vibrating sample magnetometer (VSM).

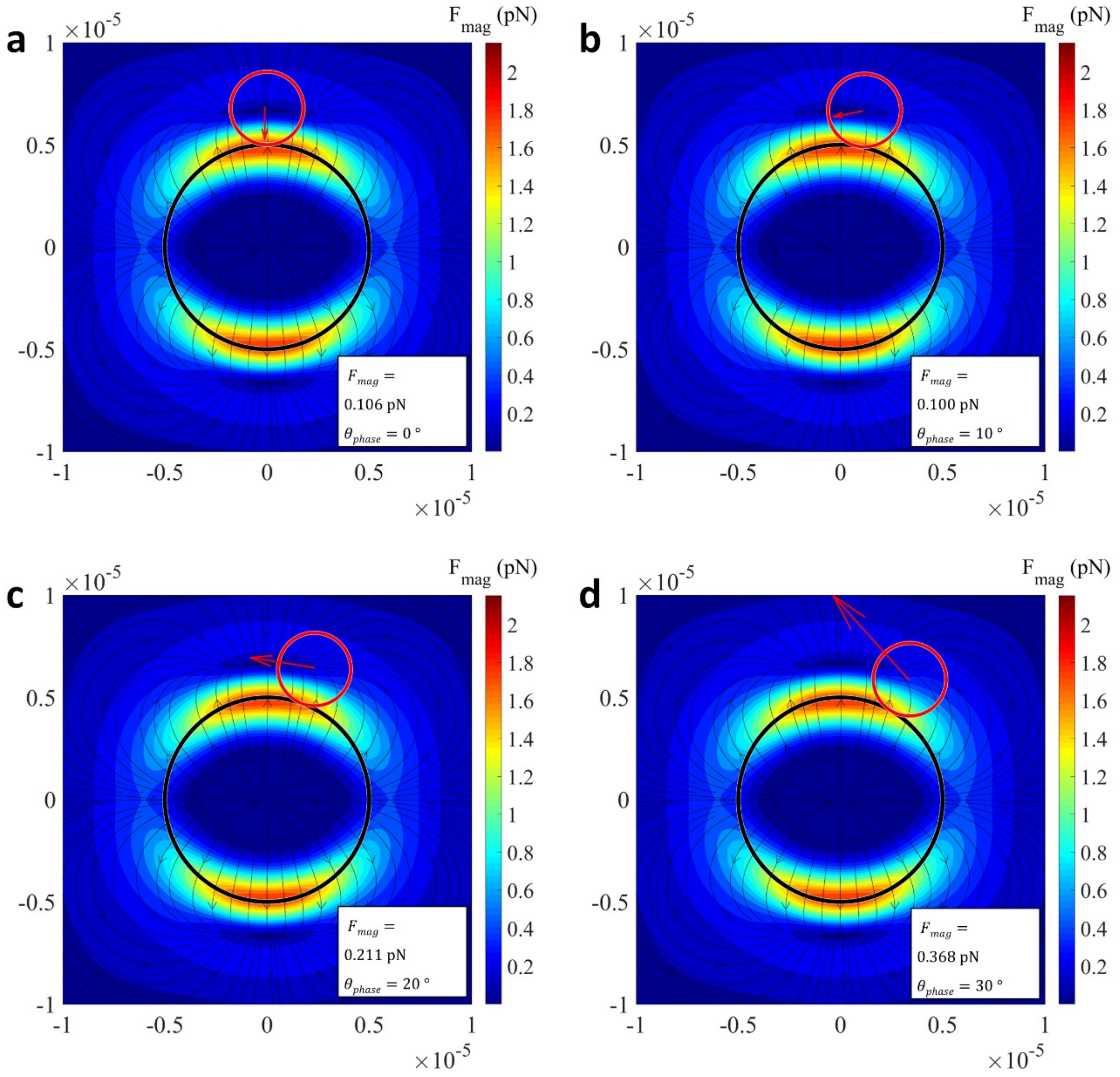

Supplementary Figure 4. The magnetic force calculations of PsD holes on negative micro-magnetic disk pattern. a−d The magnetic force is calculated to be 0.106 pN, 0.100 pN, 0.211 pN and 0.368 pN at different field rotation angles ɵ = 0°, 10°, 20° and 30° respectively. The viscous force is calculated to be 0.043 pN. The magnetic force is calculated with a ferrolfuid magnetization per field $\mathbf{M_f}/\mathbf{H}$ = 0.000376.

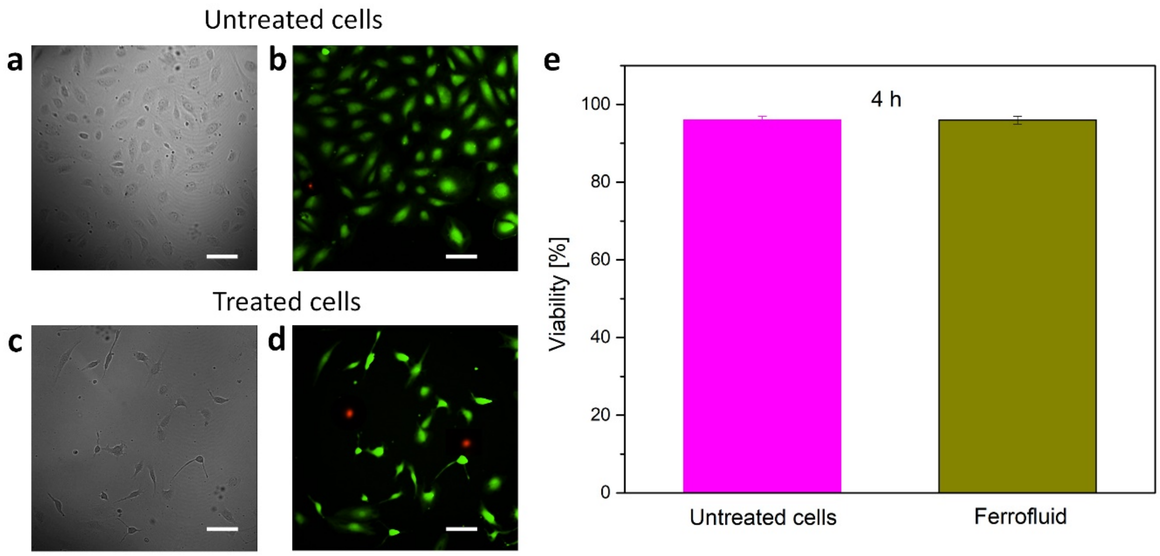

Supplementary Figure 5. Cell viability of HUVEC cells. a, b Differential interference contrast (DIC), live (green) and dead (red) fluorescence optical microscope images of HUVEC cells in the cell culture medium without ferrofluid treatment. **c, d** DIC, live (green) and dead (red) fluorescence images of HUVEC cells after 4 hours of treatment with ferrofluid (0.2% v/v). Scale bar: 100 µm. **e** Quantitative analysis of the viability of HUVEC cells demonstrating no acute toxicity induced by ferrofluid. Data are presented as the mean ± standard deviation.

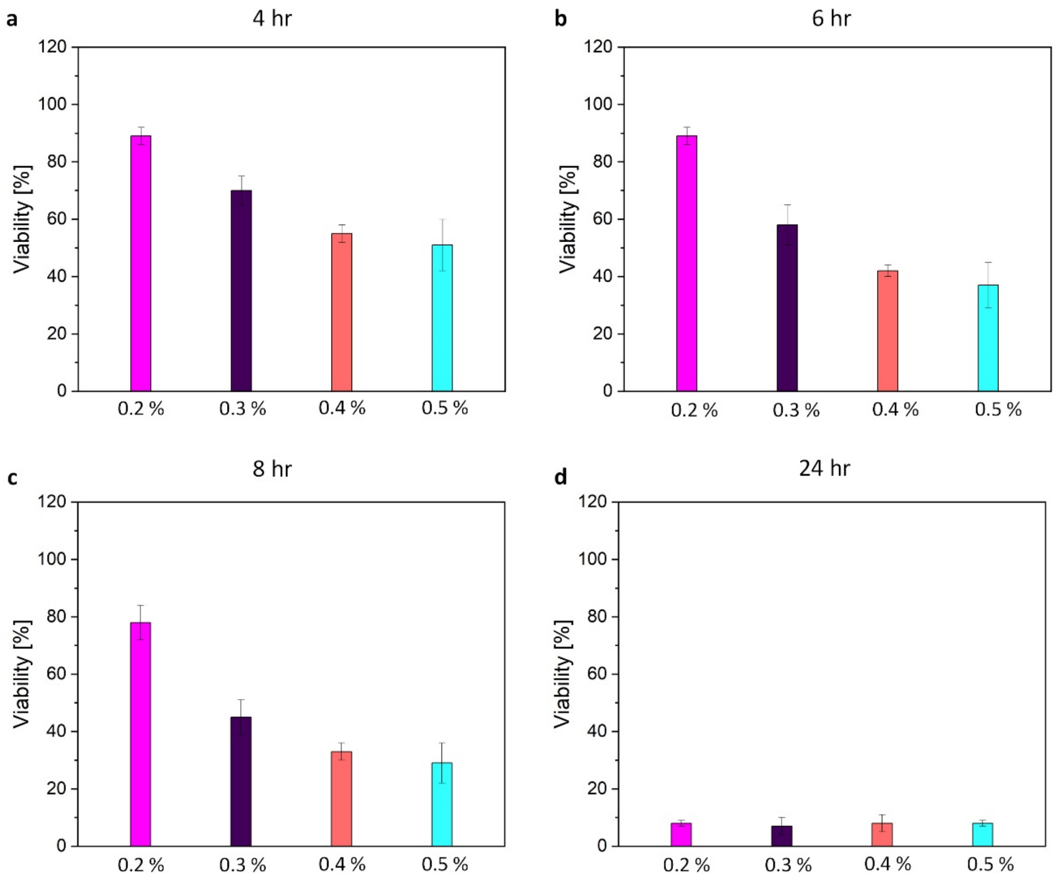

Supplementary Figure 6. Quantitative analysis of the viability of THP-1 cells in the presence of different ferrofluid concentrations under different periods of time. a−d The cells demonstrating no acute toxicity for lower ferrofluid concentrations, specifically 0.2 % v/v. Longer treatment periods for 6 hr, 8 hr and 24 hr progressively reduce the viability of cells. Data are presented as the mean ± standard deviation.

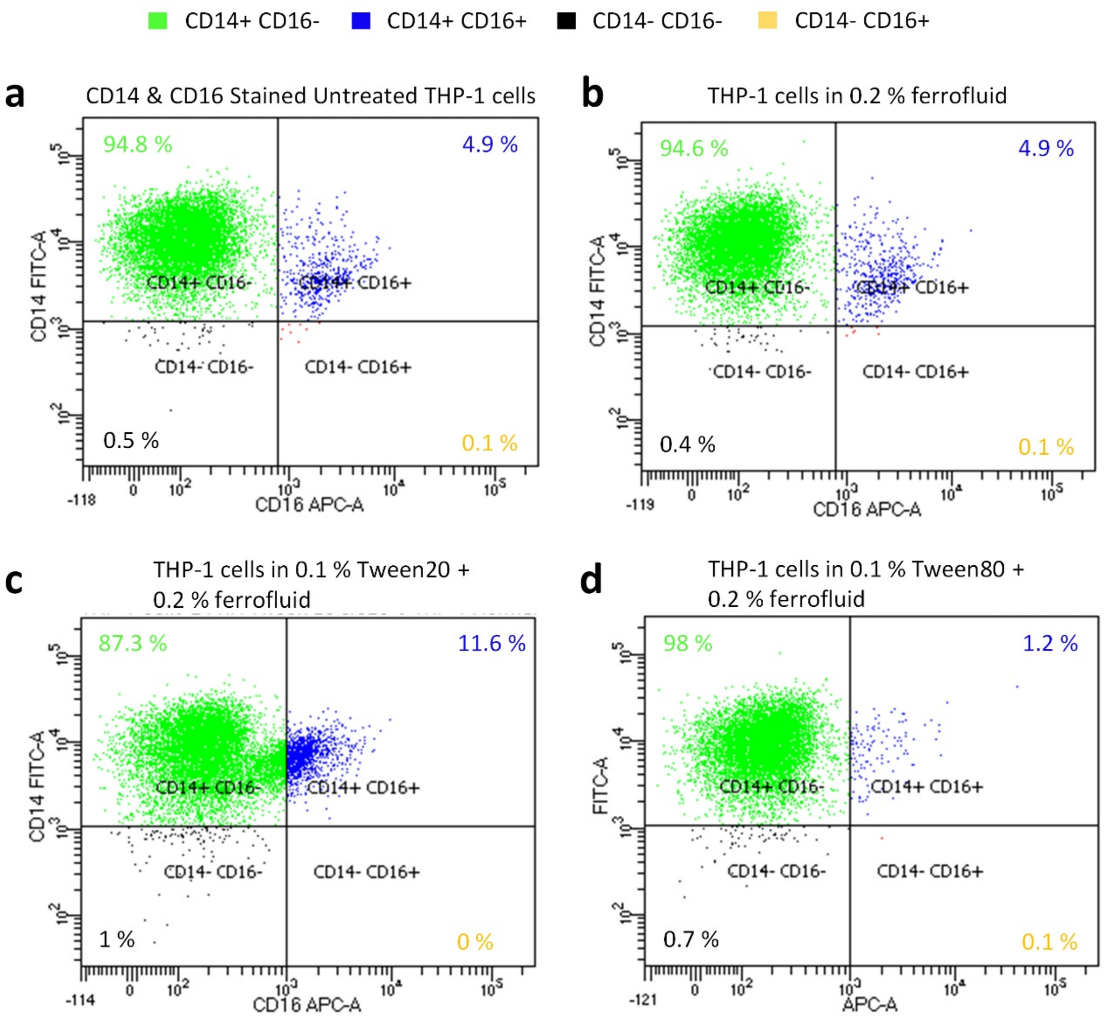

Supplementary Figure 7. Screening of cellular markers of THP-1 cells. a After 24 hr, untreated cells were stained with CD14 and CD16 markers and analysed by FACS to evaluate the expression of each marker. **b-d** Markers of THP-1 cells were analysed after 24 hr in the presence of 0.2 % v/v ferrofluid, 0.1 % Tween 20 and Tween 80 mixed with 0.2 % v/v ferrofluid, respectively.

Supplementary Note 2: Characterization of the cell marker expression.

Concerning the characteristic marker expression, we performed a fluorescence-activated cell sorting (FACS) assay for THP-1 cells, and followed CD14 and CD16 markers, characteristic monocyte/macrophage markers, on the cell surface. We did this assay by testing 4 groups of cell populations. In addition to untreated cells (Fig. S7a) and ferrofluid-treated cells as shown in Fig. S7b (with the same concentration of ferrofluid as we used in the cell separation assays), we included Tween 20 (Fig. S7c) and Tween 80 (Fig. S7d) into ferrofluid as additional non-ionic surfactants. The FACS analysis at 24 h, which is typically already too long for cell sorting assays, suggests that the concentration of ferrofluid that we use does not lead to a disparity in the expression of CD14 and CD16 markers within the population. Notwithstanding, the presence of additionally added Tween 20 to the ferrofluid solution favors CD14+ CD16+ cell population, and the presence of additionally added Tween 80 depletes this both positive cell population. Although our specific material formulation did not lead to a change in the CD14 and CD16 cell markers of THP-1 cells, the change upon additionally added surfactants suggests that the execution of a specific cell-sorting assay may require a pre-screening test for the compatibility with the surrounding materials. This having said, a similar challenge is present for all cell sorting assays that require the use of a solution other than the one where cells remain in their native environments.

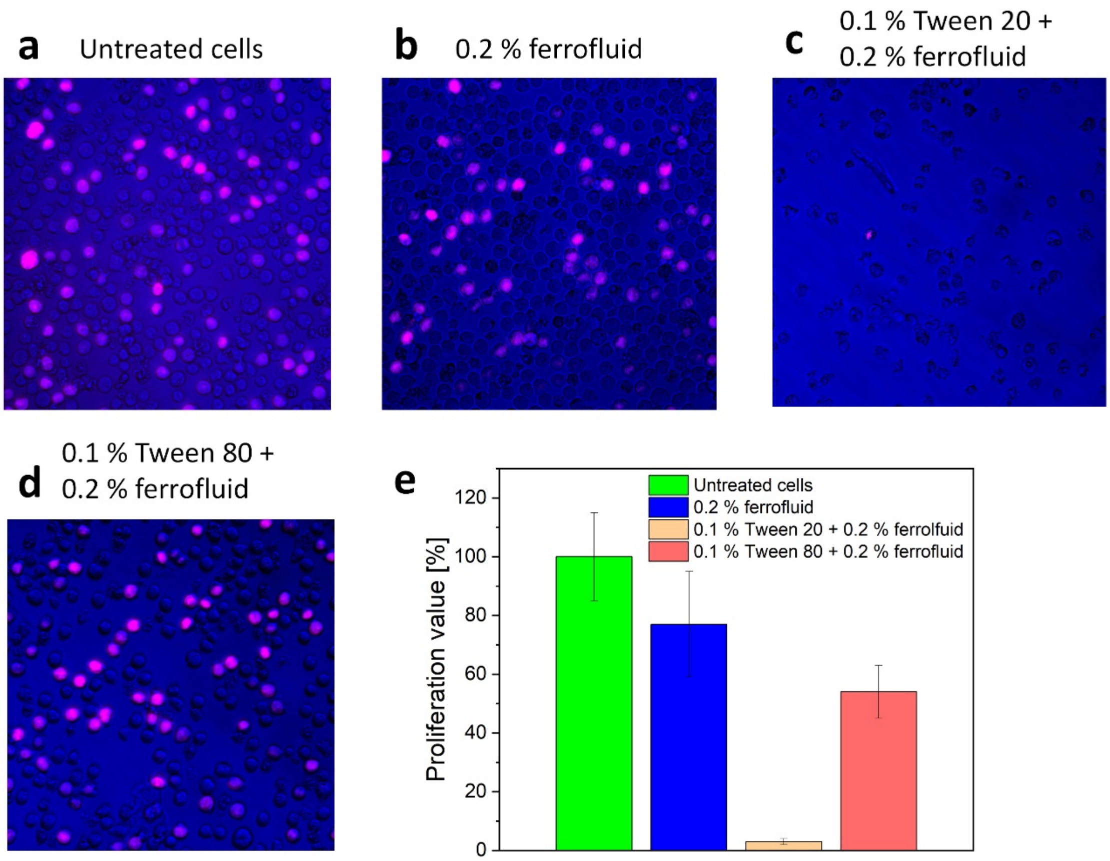

Supplementary Figure 8. Proliferation of THP-1 cells. a THP-1 cells without ferrofluid treatment. **b** Proliferation of THP-1 cells in the presence of 0.2 % v/v ferrofluid concentration. **c, d** 0.2 % v/v ferrofluid mixed with the 0.1 % Tween 20 and Tween 80, respectively. **e** Quantitative analysis of cell proliferation value under cells without ferrofluid treatment and the treatment with the ferrofluids and Tween 20 and Tween 80 at 24 hr. (Pink color: EdU incorporated (proliferative) cells).

Supplementary Note 3: Characterization of the cell proliferation.

Concerning the impact of the ferrofluid suspension on the cell proliferation, we employed a cell proliferation assay that was based on the incorporation of thymine nucleotide analog EdU to the DNA followed by fluorescence tagging of the proliferated cells. The fluorescence images show that the presence of ferrofluid reduces the rate of proliferation from normalized 100 ± 15 % to 77 ± 18 %. While we did not see a statistical significance, the reduced proliferation rate might suggest a mild acute cellular stress that was caused by the ferrofluid-containing medium in the cellular environment. Taken both cellular marker analysis and proliferation assays into account together, the cellular response to the ferrofluid overall does not detriment the utility of the developed label-free cell separation technique. While we investigated cell-ferrofluid interactions for 24 h, realistically, a cell sorting method needs to be completed within few hours to be practical. In this practical time window, cell-medium interactions seem convincingly friendly. Nevertheless, for each cell population, biocompatibility analysis needs to be a prerequisite, as cell-to-cell variations can always potentially result in severe toxic consequences.

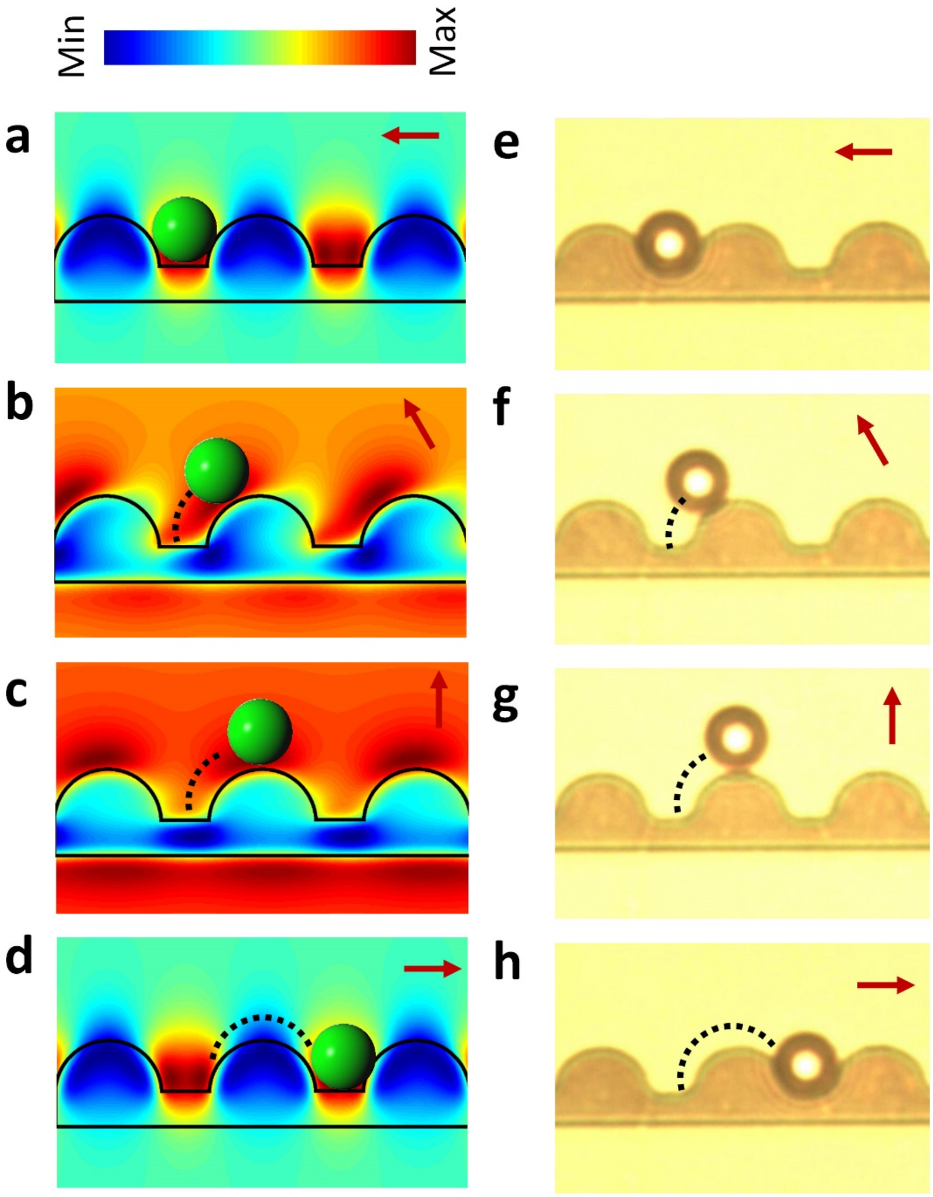

Supplementary Figure 9. Magnetic field potential energy landscapes correspond to traces of 6.72 μm PsD holes over a negative micromagnetic conductor pathways. a−d Movement of PsD holes along potential energy maxima (Red) during clockwise field rotation at angles ϴ = 180°, 120°, 60° and 0°, respectively. **e−h** Experimental traces of PsD holes on conductor pathways. Black arrows denote the trajectories of PsD holes. Red arrows represent the direction of the magnetic field.

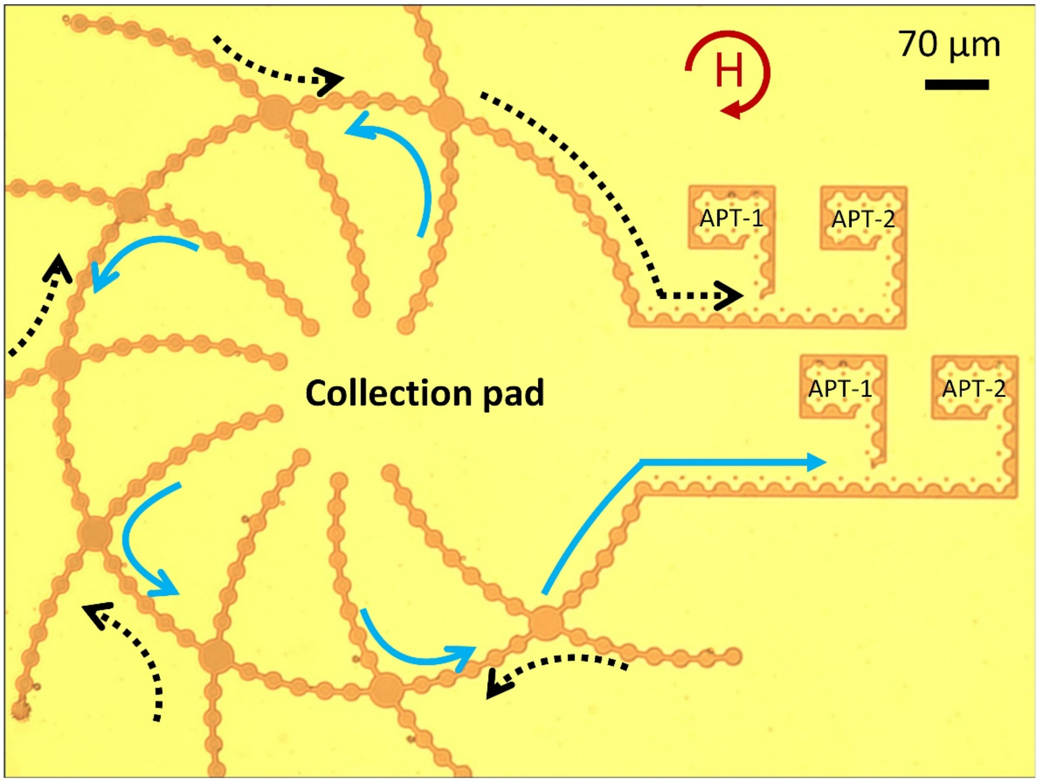

Supplementary Figure 10. **Optical image of the dispersed PsD holes on the collection pad.** Black and blue arrows denote the PsD holes path trajectories towards the compartments APT-1 and APT-2.

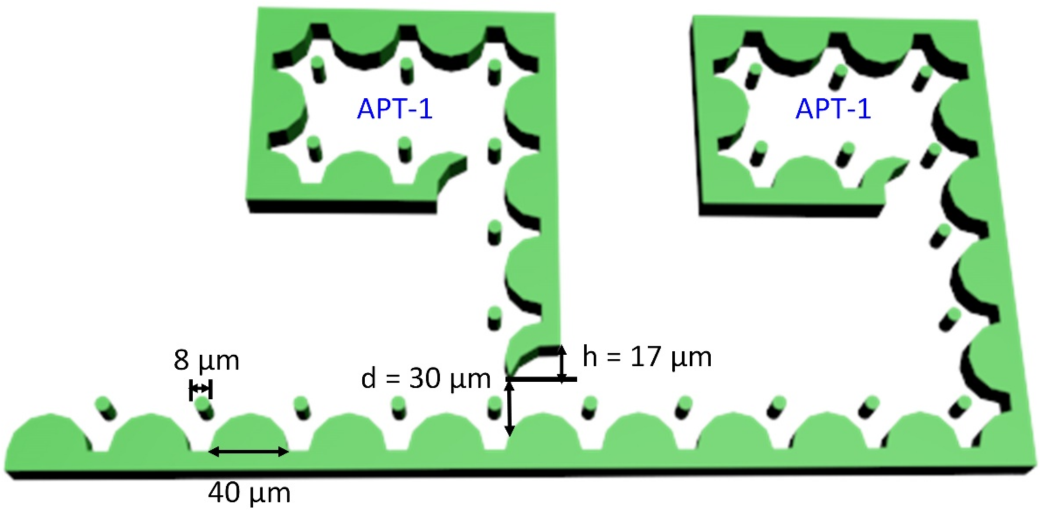

Supplementary Figure 11. Schematic diagram representing geometrical parameters of square-shaped compartments for the local separation and storage of multiple label-free THP-1cells. The diameters of half disk pattern and anti-dot are 40 µm and 8 µm respectively. The junction gap (d) and the eclipse height (h) are chosen to be 30 µm and 17 µm, respectively.

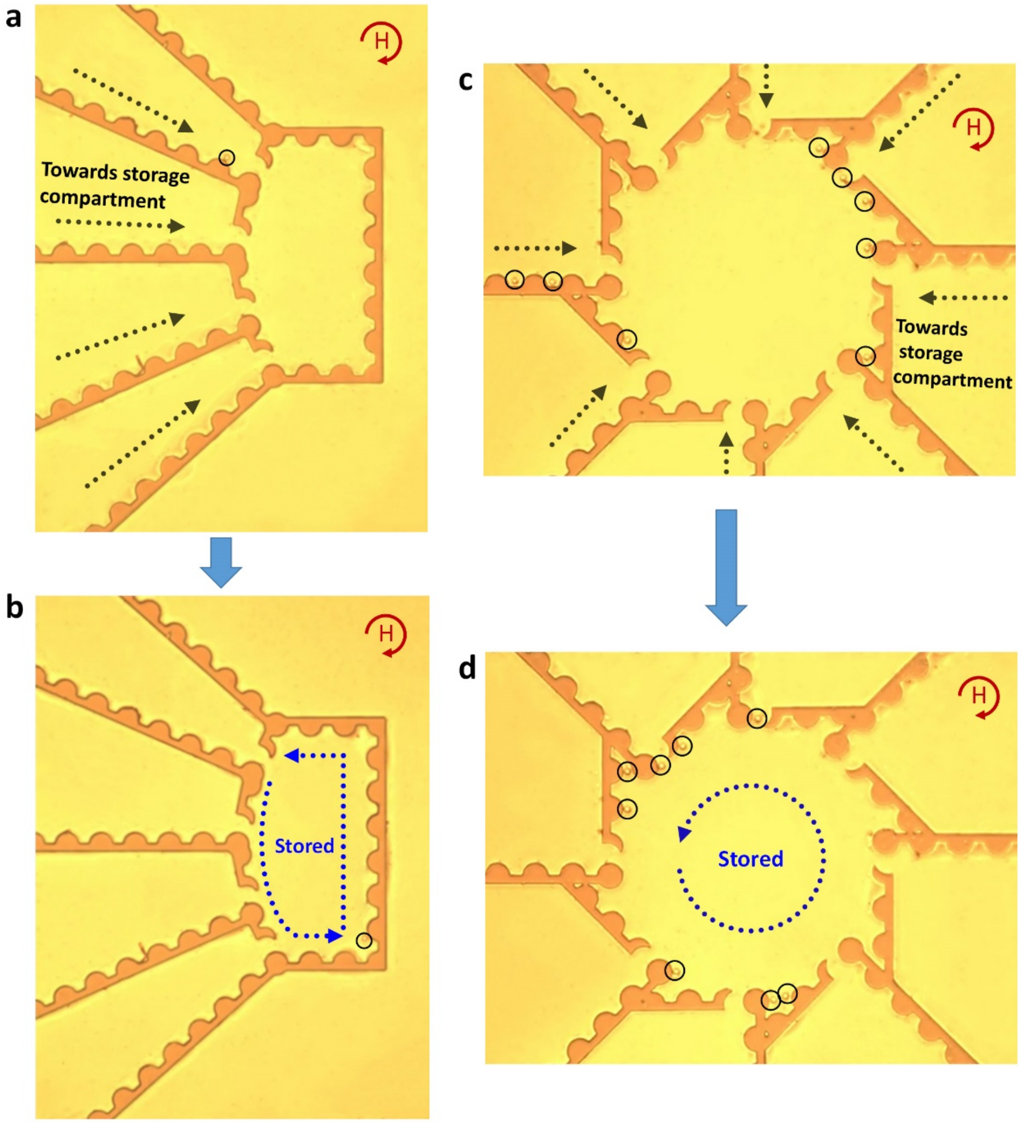

Supplementary Figure 12. Optical images of the dispersed 3.57 µm PsD holes on the capacitor micro-magnetic patterns. a, c All the PsD holes approaching towards the storage compartment. **b, d** PsD holes crossed the eclipse junction in forward bias (Mode 1). The entered PsD holes are allowed to move only inside the storage compartment under clockwise rotating magnetic fields which leads to their permanent storage. The black and blue arrow denote the path of the PsD holes.

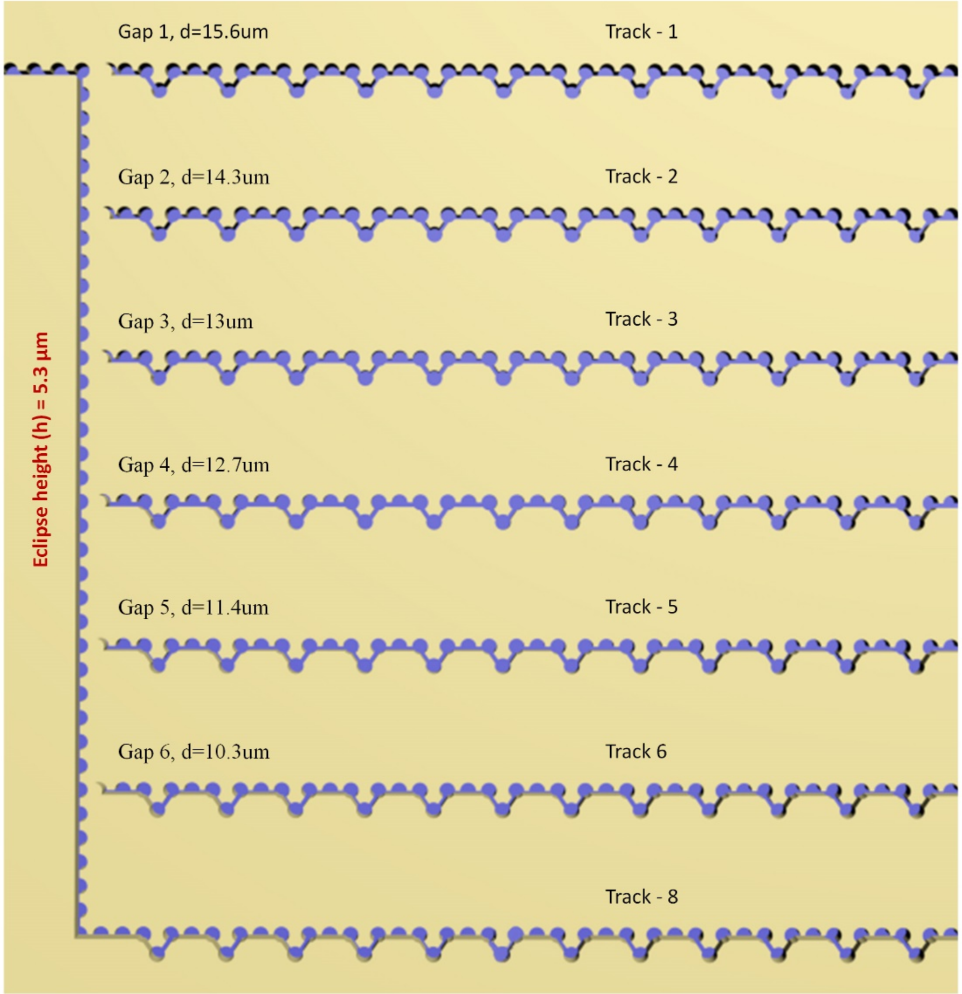

Supplementary Figure 13. Schematic diagram representing an integrated platform composed of a conductor, diode (without anti-dot), and capacitor for the synchronous sorting and storage of single PsD holes in each individual compartments. The storage compartments are designed in multiple tracks in which Track-2 is designed for 6.72 μm PsD holes and Track-3 is for 3.57 μm PsD holes.

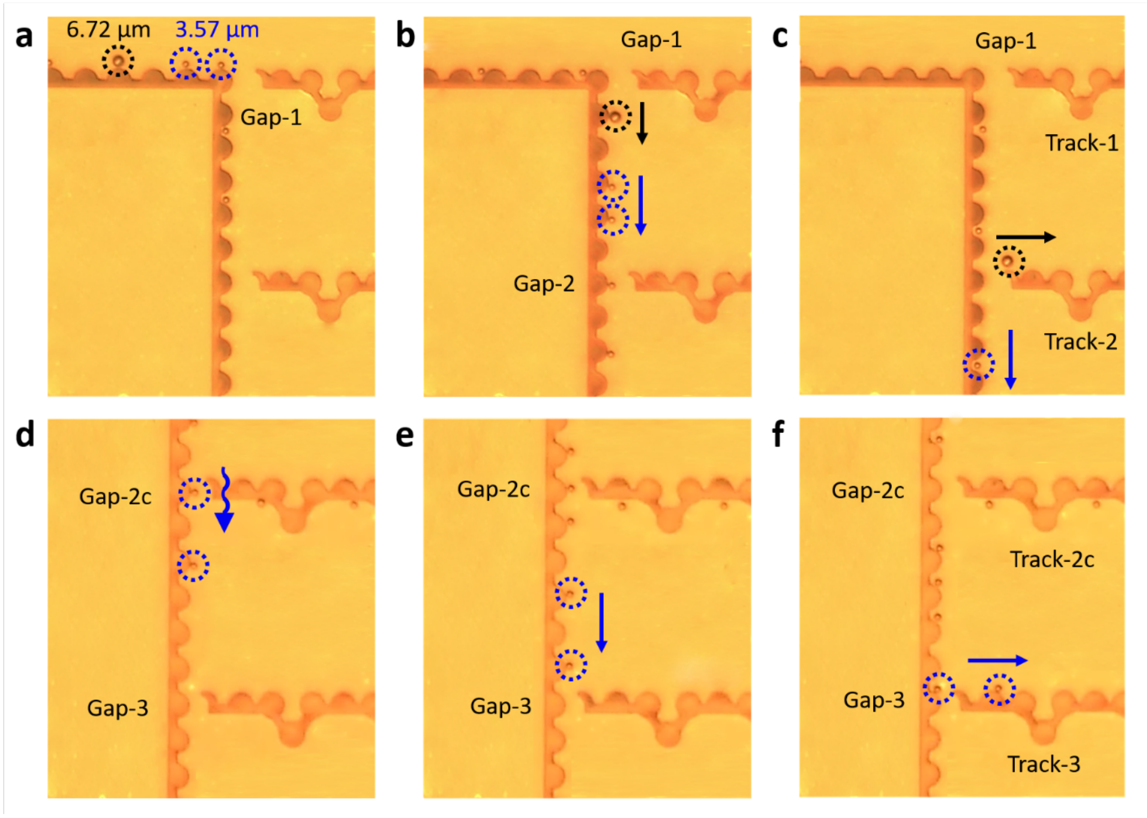

Supplementary Figure 14. Sorting of PsD holes in reverse bias mode across an eclipse diode along the distribution tracks. a, b 6.72 µm and 3.57 µm PsD holes cross the eclipse junction at Gap-1 (d = 15.6 µm). **c** Separation of 6.72 µm and 3.57 µm PsD holes by eclipse diode, where 6.72 µm PsD holes enter Track-2 due to the lack of current flow in reverse bias mode. **d-f** 3.57 µm PsD holes cross Gap-2c (d = 10.4 µm) and then navigate towards Gap-3 (d = 9.1 µm) to occupy individual storage compartments in Track-3.

Method	Precision (µm)	Labeling and Surface biomarker information	Programmability				Potential applications	Ref
			Required distance for separation	Cell control complexity	Simultaneous manipulation of cells	Automation without external processing		
Flow cytometry	Stochastic (10 ~ 20)	Required	cm	Uni-directional	Multiple (~12000 cells/s)	No	downstream assay, single cell analysis, biomarker detection	11-13
Photophoresis (fluidics)	Precise (2 ~ 10)	Not required	mm to cm	Uni-directional	Multiple (~100 cells/s)	Yes	Cell manipulation & mechanics	14,15, 17, 18
Optical tweezer	Precise (0.6 ~ 10)	Not required	below mm	Mult-idirectional	single/few	No	Cell manipulation & mechanics	14, 16,19
Acoustophoresis (fluidics)	Stochastic (12 ~ 16)	Not required	mm to cm	Uni-directional	Multiple (~1200 cells/s)	Yes	Cell manipulation	20, 22-25
Acoustic tweezer	Precise (2 ~ 10)	Not required	below mm	Multi-directional	single/few	No	Cell manipulation	21, 26
Fluidics	Stochastic (10 ~ 45)	Not required	cm	Uni-directional	Multiple (~200 cells/s)	Yes	Cell isolation	61-63
Dielectrophoresis	Precise (10 ~ 15)	Not required	mm to cm	Uni-directional	Multiple (~2400 cells/s)	Yes	Cell manipulation & selection	26-29
Positive magnetophoresis (bulk magnet)	Stochastic (3 ~ 15)	Required	mm to cm	Uni-directional	Multiple (~100 cells/s)	Yes	Cell manipulation & selection	31, 32 46, 47
Magnetic tweezer	Precise (1 ~ 5.5)	Required	None	Multi-directional	Single	No	Cell manipulation	32, 33
Positive magnetophoresis (patterned magnet)	Precise (3 ~ 15)	Required	below mm	Multi-directional	Multiple (~100 cells/s)	Yes	Cell manipulation Integration of other method	5, 6 38-43
Negative magnetophoresis (bulk magnet)	Stochastic (6 ~ 16)	Not required	mm to cm	Uni-directional	Multiple (~1200 cells/s)	Yes	Bulk cell manipulation & separation	64-66
This work	Precise (2 ~ 8)	Not required	below mm	Mult-idirectional	Multiple (500 to 8000 cells/s)	Yes	Multiplex cell control & storage of selected cells	

Supplementary Table 1: Comparison of different microfluidic methods and this work for cell manipulation.
