# Supplementary material for: Mattertronics for programmable manipulation and multiplex storage of pseudo-diamagnetic holes and label-free cells
Source: Nat Commun. 2021 May 21;12:3024. doi: 10.1038/s41467-021-23251-4 (PMC8139950; doi:10.1038/s41467-021-23251-4)
Supplement: Supplementary file 2 — Description of Additional Supplementary Files [file 41467_2021_23251_MOESM2_ESM.pdf]

Description of Additional Supplementary Files

Supplementary Movie 1.

Description: Manipulation of magnetic particles and PsD holes on the positive and negative micromagnetic disk patterns.

Supplementary Movie 2.

Description: Directional control of single 6.72 μm PsD hole on the negative micromagnetic conductor pattern.

Supplementary Movie 3.

Description: Switching of single 3.57 μm PsD hole on the negative micromagnetic eclipse diode patterns.

Supplementary Movie 4.

Description: Separation and storage of multiple label-free THP-1 cells in the square-shaped compartments.

Supplementary Movie 5.

Description: Individual storage capacitors for single PsD holes.

Supplementary Movie 6.

Description: Magnetic storage tracks for parallel processing of single PsD holes. The designed eclipse junction allows the passage of PsD holes from the lower track to the upper track resulting in the efficient distribution of single cells without clogging and resulting in high throughput.
