# Supplementary material for: Mattertronics for programmable manipulation and multiplex storage of pseudo-diamagnetic holes and label-free cells
Source: Nat Commun. 2021 May 21;12:3024. doi: 10.1038/s41467-021-23251-4 (PMC8139950; doi:10.1038/s41467-021-23251-4)
Supplement: Supplementary file 9 — Reporting Summary [file 41467_2021_23251_MOESM9_ESM.pdf]

nature research

Corresponding author(s): NCOMMS-20-22800A

Last updated by author(s): Mar 9, 2021

Reporting Summary

Nature Research wishes to improve the reproducibility of the work that we publish. This form provides structure for consistency and transparency in reporting. For further information on Nature Research policies, see our Editorial Policies and the Editorial Policy Checklist.

Statistics

For all statistical analyses, confirm that the following items are present in the figure legend, table legend, main text, or Methods section.

n/a	Confirmed	
☒	☐	The exact sample size (n) for each experimental group/condition, given as a discrete number and unit of measurement
☒	☐	A statement on whether measurements were taken from distinct samples or whether the same sample was measured repeatedly
☒	☐	The statistical test(s) used AND whether they are one- or two-sided<br>*Only common tests should be described solely by name; describe more complex techniques in the Methods section.*
☒	☐	A description of all covariates tested
☒	☐	A description of any assumptions or corrections, such as tests of normality and adjustment for multiple comparisons
☒	☐	A full description of the statistical parameters including central tendency (e.g. means) or other basic estimates (e.g. regression coefficient) AND variation (e.g. standard deviation) or associated estimates of uncertainty (e.g. confidence intervals)
☒	☐	For null hypothesis testing, the test statistic (e.g. F, t, r) with confidence intervals, effect sizes, degrees of freedom and P value noted<br>*Give P values as exact values whenever suitable.*
☒	☐	For Bayesian analysis, information on the choice of priors and Markov chain Monte Carlo settings
☒	☐	For hierarchical and complex designs, identification of the appropriate level for tests and full reporting of outcomes
☒	☐	Estimates of effect sizes (e.g. Cohen's d, Pearson's r), indicating how they were calculated

Our web collection on statistics for biologists contains articles on many of the points above.

Software and code

Policy information about availability of computer code

Data collection	The Mumax3 and MATLAB codes used in this study are available from the corresponding author on request.
Data analysis	The magnetic hysteresis measurements of ferrofluids were performed using a Lake-shore 7400 series vibrating sample magnetometer (VSM). Scanning electron microscopy Images of micro-magnetic patterns were obtained using Hitachi Co., model S-4800, at DGIST. Transmission electron microscopy Images of ferrofluids were achieved by Hitachi Co., model HF-3300, at DGIST. Particle motions were tracked by video microscopy using an IMC-1040FT video camera.

For manuscripts utilizing custom algorithms or software that are central to the research but not yet described in published literature, software must be made available to editors and reviewers. We strongly encourage code deposition in a community repository (e.g. GitHub). See the Nature Research guidelines for submitting code & software for further information.

Data

Policy information about availability of data

All manuscripts must include a data availability statement. This statement should provide the following information, where applicable:

- Accession codes, unique identifiers, or web links for publicly available datasets
- A list of figures that have associated raw data
- A description of any restrictions on data availability

All data generated or analyzed during this study are included in the published article and its Supplementary Information and are available from the corresponding author on reasonable request.

Field-specific reporting

Please select the one below that is the best fit for your research. If you are not sure, read the appropriate sections before making your selection.

[x] Life sciences   [ ] Behavioural & social sciences   [ ] Ecological, evolutionary & environmental sciences

For a reference copy of the document with all sections, see nature.com/documents/nr-reporting-summary-flat.pdf

Life sciences study design

All studies must disclose on these points even when the disclosure is negative.

Sample size	4 technical replicates for each group within each independent repeat.
Data exclusions	No data were excluded.
Replication	Each experiment that contained cells were replicated at least 3 times independently.
Randomization	Not applicable
Blinding	No blinding was applied.

Reporting for specific materials, systems and methods

We require information from authors about some types of materials, experimental systems and methods used in many studies. Here, indicate whether each material, system or method listed is relevant to your study. If you are not sure if a list item applies to your research, read the appropriate section before selecting a response.

Materials & experimental systems

n/a	Involved in the study	
[ ]	[x]	Antibodies
[ ]	[x]	Eukaryotic cell lines
[x]	[ ]	Palaeontology and archaeology
[x]	[ ]	Animals and other organisms
[x]	[ ]	Human research participants
[x]	[ ]	Clinical data
[x]	[ ]	Dual use research of concern

Methods

n/a	Involved in the study	
[x]	[ ]	ChIP-seq
[ ]	[x]	Flow cytometry
[x]	[ ]	MRI-based neuroimaging

Antibodies

Antibodies used	Cd14: Mouse anti Human, FITC, Clone: M5E2, BD (Catalog number 555397). Cd16: APC Mouse Anti-Human, Clone B73.1, BD (Catalog number 561304)
Validation	Cd14: Validated by BD (Human (QC Testing) Rhesus, Cynomolgus, Baboon, Dog (Tested in Development)). Cd16: Human (QC Testing)

Eukaryotic cell lines

Policy information about cell lines

Cell line source(s)	THP-1 (DSMZ: ACC 16)

Authentication

Performed by DSMZ: (1) multiplex PCR of minisatellite markers revealed a unique DNA profile, (2) originally confirmed as human with IEF of AST, MDH, NP, and recently by STR typing, (3) Cytogenesis: human near-tetraploid karyotype - 94(88-96) <4n>XY/XXY, -Y, +1, +3, +6, +6, -8, -13, -19, -22, -22, +2mar, add(1)(p11), del(1)(q42.2), i(2q), del(6)(p21)x2-4, i(7p), der(9)t(9;11)(p22;q23)i(9)(p10)x2, der(11)t(9;11)(p22;q23)x2, add(12)(q24)x1-2, der(13)t(8;13)(p11;p12), add(?18)(q21) - carries t(9;11) associated with AML M5, (4) Molecular genetics: expression of fusion gene KMT2A-MLLT3 (MLL-MLLT3; MLL-AF9) confirmed by RT-PCR

Mycoplasma contamination

Confirmed by DMSZ. We did further confirmation by immunocytochemistry staining of the nuclei.

Commonly misidentified lines
(See ICLAC register)

Name any commonly misidentified cell lines used in the study and provide a rationale for their use.

Flow Cytometry

Plots

Confirm that:

- [x] The axis labels state the marker and fluorochrome used (e.g. CD4-FITC).
- [x] The axis scales are clearly visible. Include numbers along axes only for bottom left plot of group (a 'group' is an analysis of identical markers).
- [x] All plots are contour plots with outliers or pseudocolor plots.
- [x] A numerical value for number of cells or percentage (with statistics) is provided.

Methodology

Sample preparation

Several samples of THP-1 cells incubated at 1 million cells with different concentrations of Ferro Fluid for 24 hours. After exposure the cells were rinsed by centrifugation and sterile DPBS. A correct addition of fluorescent surface markers for CD14 and CD16 were added to all samples and incubated at room temperature for 30 minutes. A series of washes was performed before being suspended at 1 million cells per 1 mL of FAC's buffer solution. The cells were then immediately analyzed on our BD LSRFortessa X20.

Instrument

BD LSRFortessa X20

Software

BD FACSDiva

Cell population abundance

1 million cells in 1 mL

Gating strategy

To ascertain that the cells were stained positive for the particular surface marker (e.g. CD16) in test samples all the two surface markers were combined and the samples were acquired using the same voltage settings and compensation parameters as done for a combination of their respective controls. 5000 events were analyzed by measurement and separated according to their CD16/CD14 expression profile on the cell surface in post acquisition analysis. THP1 cells were gated based on forward scatter and side scatter characteristics. In addition, THP1 cells were defined according to their CD14 and CD16 expression profile.

- [x] Tick this box to confirm that a figure exemplifying the gating strategy is provided in the Supplementary Information.
